# CircGSK3β mediates PD-L1 transcription through miR-338-3p/PRMT5/H3K4me3 to promote breast cancer cell immune evasion and tumor progression

**DOI:** 10.1038/s41420-024-02197-8

**Published:** 2024-10-04

**Authors:** Lin Liang, Mengxiang Gao, Wentao Li, Jingqiong Tang, Qian He, Feng Zeng, Jiaying Cao, Siyi Liu, Yan Chen, Xin Li, Yanhong Zhou

**Affiliations:** 1 Breast Cancer Center, Department of General Surgery, Xiangya Hospital, Central South University, Changsha, Hunan 410008 China; 2 National Clinical Research Center of Geriatric Disorders, Xiangya Hospital of Central South University, Changsha, Hunan 410008 China; 3 Cancer Research Institute, Basic School of Medicine, Central South University, Changsha, Hunan 410011 China; 4 Department of Geriatrics, The Second Xiangya Hospital of Central South University, Changsha, Hunan 410011 China; 5 Department of Radiation Oncology, Hunan Cancer Hospital & the Afliated Cancer Hospital of Xiangya School of Medicine, Central South University, Changsha, Hunan 410013 China

**Keywords:** Immunoediting, Breast cancer

## Abstract

Circular RNA (circRNA) plays a pivotal role in breast cancer onset and progression. Understanding the biological functions and underlying molecular mechanisms of dysregulated circRNAs in breast cancer is crucial for elucidating its pathogenesis and identifying potential therapeutic targets. In this study, we investigated the role and molecular mechanism of circGSK3β in breast cancer. We found that circGSK3β is highly expressed in breast cancer cell lines, where it promotes cell proliferation, migration, and invasion, thereby driving breast cancer progression. Furthermore, we observed a close association between circGSK3β expression levels and immune evasion in breast cancer cells. Mechanistically, circGSK3β acts as a competing endogenous RNA (ceRNA) by interacting with miR-338-3p, thereby promoting breast cancer cell proliferation, migration, and invasion. Additionally, circGSK3β positively regulates the expression of the target gene PRMT5 through its interaction with miR-338-3p. This, in turn, enhances H3K4me3 recruitment to the promoter region of PD-L1, resulting in upregulation of PD-L1 expression and consequent immune evasion in breast cancer. In summary, our findings underscore the significance of the circGSK3β-miR-338-3p-PRMT5-H3K4me3 axis in promoting breast cancer progression and immune evasion. CircGSK3β emerges as a critical player in breast cancer pathogenesis, potentially serving as a diagnostic and prognostic marker, and offering novel insights into the role of circRNAs in breast cancer progression.

## Introduction

Breast cancer (BC) stands as the foremost prevalent female cancer globally and ranks as the second most common cause of female mortality [1]. Recent statistics from the International Agency for Research on Cancer (IARC) underscore the alarming reality that an estimated 2.26 million women will receive a BC diagnosis worldwide in 2020, representing 11.7% of all cancer diagnoses and surpassing lung cancer as the most prevalent cancer globally [2]. Despite significant strides in diagnostic techniques and comprehensive treatments, a substantial number of patients succumb to cancer metastasis, underscoring the imperative of delving into the molecular underpinnings of BC initiation and progression. Identifying novel molecular targets and devising innovative therapeutic strategies hold the promise of enhancing the prognosis for patients with advanced BC [3, 4].

Circular RNAs (circRNAs) constitute a class of RNA molecules distinguished by their covalently closed loop structure devoid of 5’ to 3’ polarity or polyadenylated tails [5]. Generated via reverse splicing of precursor mRNA exons, circRNAs predominantly localize within the cytoplasm [6–8]. A mounting body of evidence underscores the involvement of circRNAs in diverse physiological and pathological processes by modulating gene expression, apoptosis, cell cycle progression, migration, invasion, and immune evasion [9, 10]. For instance, circEZH2 has been shown to significantly enhance colorectal cancer cell proliferation and migration both in vitro and in vivo by modulating the miR-133b/IGF2BP2/CREB1 regulatory axis [11]. On the other hand, circEZH2 could reverse post-transcriptional repression of KLF5 by sponging miR-217-5p, thereby accelerating CXCR4-induced EMT in BC. KLF5 transcriptional activation of FUS promotes the back-splicing process of circEZH2, leading to a FUS/circEZH2/KLF5/CXCR4 positive feedback loop [12]. Similarly, circCD44 drives the progression of triple-negative breast cancer (TNBC) via the miR-502-5p/KRAS and IGF2BP2/C-Myc signaling pathways, thereby emerging as a potential therapeutic target for TNBC [13]. CircMET has been implicated in hepatocellular carcinoma development and immune tolerance through the Snail/DPP4/CXCL10 axis [14]. Additionally, circDLG1 acts as a miRNA sponge for miR-141-3p, augmenting the expression of the miR-141-3p target gene chemokine 12 (CXCL12) to promote gastric cancer progression and immune evasion [15]. Notably, recent research has unveiled the role of circRNA-circGSK3B in hepatocellular carcinoma, where it promotes cell proliferation, migration, and invasion by sponging miR-1265 and regulating CAB39 expression [16]. Moreover, circGSK3B has been shown to interact with EZH2, impeding its binding to the RORA promoter and resulting in elevated RORA expression, thereby restraining tumor progression [17]. Despite emerging insights into the aberrant pathways associated with circGSK3β and BC, the precise mechanism underlying circGSK3β-mediated BC progression and immune evasion remains incompletely elucidated, necessitating further comprehensive investigation.

Protein arginine methyltransferases (PRMTs) represent a class of methyltransferases featuring a group 7β-strand structure responsible for methylating proteins on arginine residues [18]. Arginine-mediated methylation stands as the predominant form of protein methylation in mammalian cells, exerting regulatory roles in signal transduction, RNA processing, chromatin stability, and transcriptional regulation [19]. Among PRMT family members, Protein arginine methyltransferase 5 (PRMT5) stands out, catalyzing the transfer of methyl groups from S-adenosylmethionine (SAM) to the guanidine nitrogen of protein arginine, thereby generating methylated guanidine and S-adenosylhomocysteine (SAH) [20]. As a type II protein arginine methyltransferase, PRMT5 symmetrically dimethylates (me2s) H2AR3, H3R2, H3R8, and H4R3, frequently associated with transcriptional repression or activation [21]. Serving as a crucial epigenetic modulator, PRMT5 interacts with chromatin remodeling factors, co-repressors, and co-activators, thereby regulating the transcription of tumor suppressor genes [22]. Notably, PRMT5-mediated symmetric dimethylation of H3R2 (H3R2me2s) is recognized by WDR5 post-methylation, facilitating the recruitment of the SET1/MLL complex and subsequent histone H3K4 trimethylation (H3K4me3), thus promoting gene expression [23]. Despite its pivotal role in tumor immunology, PRMT5 has been shown to be unable to modulate PD-L1 gene transcription through histone methylation (H3R2, H3R8, and H4R3) in cervical cancer cells [24, 25]. Therefore, whether PRMT5 can regulate PD-L1 expression via H3K4me3 in BC cells remains unexplored and warrants further investigation.

In this study, we observed elevated expression of circGSK3β in BC cell lines, correlating with enhanced proliferation, migration, invasion, and subsequent tumor progression. Further investigations unveiled a significant association between circGSK3β expression and immune evasion in BC. Specifically, circGSK3β positively regulates PRMT5 expression via miR-338-3p, thereby facilitating the recruitment of H3K4me3 to the PD-L1 promoter, ultimately fostering immune escape and tumor progression in BC. These findings not only shed light on the pathogenic mechanism involving the circGSK3β-miR-338-3p-PRMT5-H3K4me3 axis in BC but also present promising therapeutic targets for BC patients.

## Results

### Characteristics of CircGSK3β in breast cancer cells

**Fig. 1:**
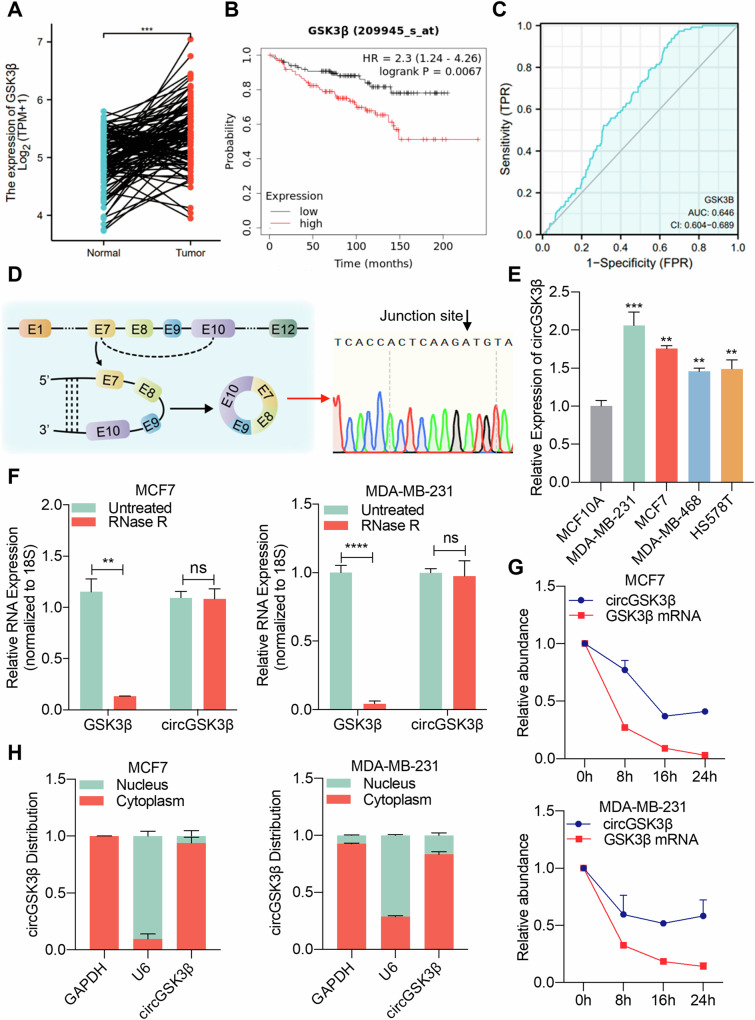
Characteristics of CircGSK3β in breast cancer cells. **A** Expression of GSK3β in 113 paired BC tissues and para-cancerous tissues, analyzed using paired samples T test, ***P < 0.001. **B** Analysis of the relationship between GSK3β expression and prognosis of BC patients using the GSE11121 dataset. **C** Receiver Operating Characteristic (ROC) curve demonstrating the clinical correlation of GSK3β. **D** Schematic diagram of CircGSK3β formation and Sanger sequencing peak diagram. **E** qRT-PCR detection of circGSK3β expression in normal breast cells and BC cell lines, **P < 0.01, ***P < 0.001. **F** qRT-PCR measuring the expression of circGSK3β and GSK3β mRNA in MDA-MB-231 and MCF7 cells treated/untreated with RNase R, using 18S as the internal reference, **P < 0.01, ****P < 0.0001, ns, not significant. **G** qRT-PCR detecting the abundance changes of GSK3β mRNA and circGSK3β in MDA-MB-231 and MCF7 cells treated with actinomycin D for 0, 8, 16, and 24 h respectively, using 18S as the internal reference. **H** Nucleocytoplasmic separation experiment detecting the localization of circGSK3β in MDA-MB-231 and MCF7 cells, and expression of circGSK3β relative to total RNA. Data are expressed as mean ± SD of at least three independent experiments.

GSK3β, an evolutionarily conserved serine/threonine kinase, plays crucial roles in regulating glycogen synthase activity, glycogen synthesis, and various aspects of tumorigenesis, invasion, and metastasis [26–28]. Through analysis of TCGA data, we observed elevated expression of GSK3β in BC tissues, with the Kaplan-Meier Plotter GSE11121 dataset revealed an association between GSK3β and poor prognosis in patients (Fig. 1A, B). Furthermore, our analysis of the ROC curve indicated that it has high clinical diagnostic value (AUC = 0.6646) (Fig. 1C). Notably, emerging evidence suggests that the functions of circular RNAs (circRNAs) in cancer are intimately linked to the oncogenic or oncostatic effects of their parent genes [29]. Among these, circGSK3β, derived from GSK3β, has been implicated in promoting cell proliferation, migration, and invasion in gastric, hepatocellular, and esophageal squamous cell carcinomas [16, 17, 30]. However, the role and underlying mechanisms of circGSK3β in BC remain unexplored. Thus, our study aims to elucidate the involvement of circGSK3β in BC and its associated mechanisms.

CircGSK3β originates from the back-splicing of exons 7-10 of the GSK3β gene, resulting in a full length of 420 nucleotides, consistent with the splicing sequence of hsa_circ_0008797 in the circbase database (Fig. 1D). To elucidate the involvement of circGSK3β in BC, we assessed its expression levels in immortalized mammary epithelial cells (MCF-10A) and various BC cell lines (MDA-MB-231, MCF7, MDA-MB-468, HS578T). Our analysis revealed upregulation of circGSK3β expression in BC cell lines compared to normal breast epithelial cells MCF-10A, with the highest expression observed in MDA-MB-231 and MCF7 cells (Fig. 1E). Therefore, we selected these two BC cell lines for further investigation into the role of circGSK3β in BC and its specific regulatory mechanisms.

RNase R, an exoribonuclease, selectively degrades linear RNA while leaving circular RNA intact. Consistently, the linear transcript of GSK3β was susceptible to degradation by RNase R, whereas the circular transcript of circGSK3β remained unaffected (Fig. 1F). Due to its circular conformation, circGSK3β exhibited greater stability compared to linear GSK3β mRNA following treatment with Actinomycin D, a transcription inhibitor, in MDA-MB-231 and MCF7 cells (Fig. 1G). The cellular localization of circGSK3β was assessed via qRT-PCR analyses, revealing predominant localization in the cytoplasm of BC cells (Fig. 1H). This suggests a potential functional role for circGSK3β in the cytoplasm of BC cells. In summary, our findings establish that circGSK3β adopts a circular structure and predominantly localizes to the cytoplasm in BC cells, laying the groundwork for further exploration of its mechanistic role.

### CircGSK3β promotes breast cancer progression

**Fig. 2:**
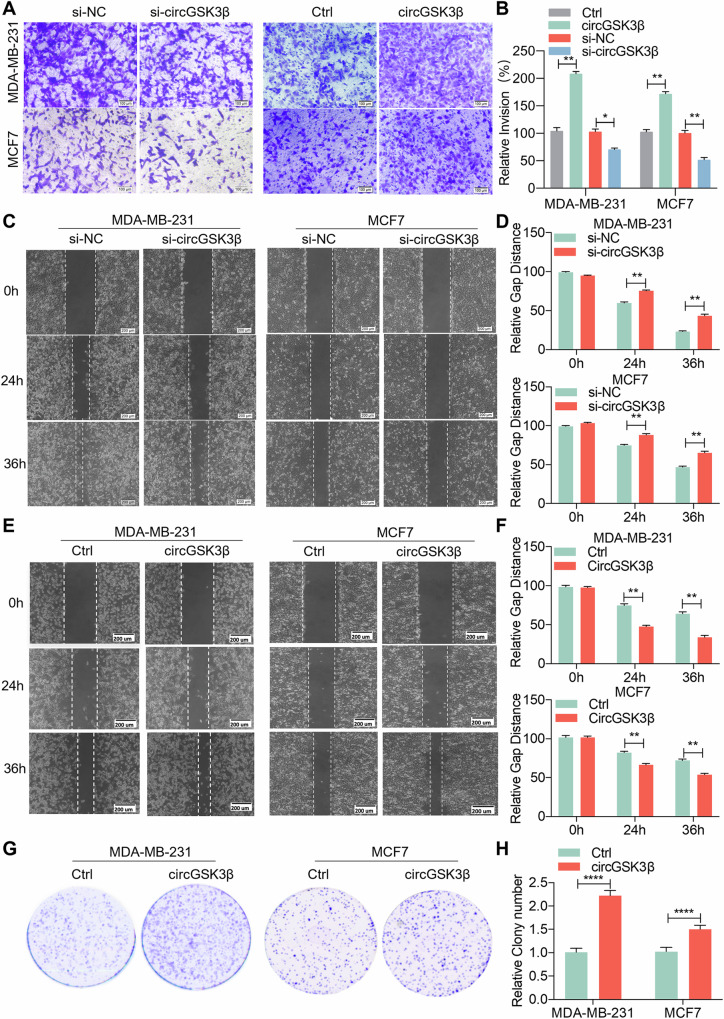
CircGSK3β promotes breast cancer progression. **A**, **B** Transwell analysis of BC cell lines MDA-MB-231 and MCF7 treated with circGSK3β overexpression/knockdown. **A** Representative image of the Transwell experiment. **B** Statistical analysis of the Transwell experiment. *P < 0.05, **P < 0.01, repeated three times. **C**, **D** Wound-healing experiments in BC cell lines MDA-MB-231 and MCF7 treated with circGSK3β knockdown. **C** Representative image of the wound-healing experiment. **D** Statistical analysis of the wound-healing experiment. **P < 0.01, repeated three times. **E**, **F** Wound-healing experiments in BC cell lines MDA-MB-231 and MCF7 treated with circGSK3β overexpression. **E** Representative image of the wound-healing experiment. **F** Statistical analysis of the wound-healing experiment. **P < 0.01, repeated three times. **G**, **H** Colony formation experiments in BC cell lines MDA-MB-231 and MCF7 treated with circGSK3β overexpression. **G** Representative image of the colony formation experiment. **H** Statistical analysis of the colony formation experiment. ****P < 0.0001, repeated three times. si-NC means control siRNA. Ctrl means the control vector for circGSK3β overexpression.

To assess the impact of circGSK3β on BC development and progression, we engineered an overexpression plasmid for circGSK3β (Supplementary Fig. S1A) and designed three circGSK3β interference sequences (si-circGSK3β 01, 02, 03) targeting the splicing site (Supplementary Figure S1B). Subsequently, we evaluated the overexpression efficiency of circGSK3β in MDA-MB-231 and MCF7 cell lines, as well as the silencing efficiency of si-circGSK3β in these cell lines, through qRT-PCR analysis (Supplementary Fig. S1C). Our Matrigel invasion assay results demonstrated that knockdown of circGSK3β reduced the invasion ability of BC cells in both MDA-MB-231 and MCF7 cell lines, whereas overexpression of circGSK3β enhanced their invasion capability (Fig. 2A, B). Furthermore, results from the scratch wound-healing assay revealed that downregulation of circGSK3β expression led to a significantly smaller wound healing area in BC cell lines MDA-MB-231 and MCF7 compared to the control group (Fig. 2C, D), whereas overexpression of circGSK3β resulted in a significantly larger wound healing area compared to the control group (Fig. 2E, F). Additionally, colony formation experiments further confirmed that circGSK3β overexpression promoted BC cell proliferation (Fig. 2G, H). In summary, our findings indicate that circGSK3β enhances the proliferation, invasion, and migration capabilities of BC cells, thereby promoting BC progression.

### CircGSK3β acts as a sponge for miR-338-3p in breast cancer and promotes breast cancer progression

**Fig. 3:**
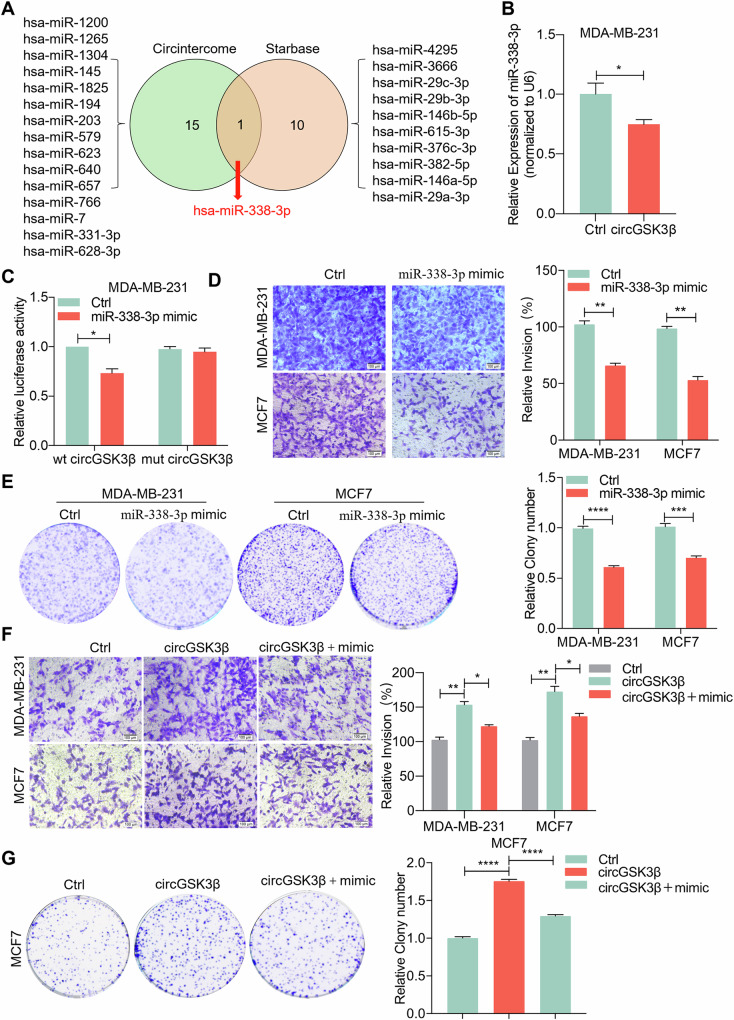
CircGSK3β acts as a sponge for miR-338-3p in breast cancer and promotes breast cancer progression. **A** Schematic diagram of miRNAs predicted by Starbase and Circinteractome websites that may bind to circGSK3β. **B** qRT-PCR detecting the expression level of miR-338-3p after overexpressing circGSK3β, *P < 0.05, repeated three times. **C** Luciferase reporter gene assay of miR-338-3p mimics and circGSK3β wild-type or mutant luciferase reporter gene co-transfected into MDA-MB-231 cells. wt: wild type; mut: mutant type; compared with Ctrl, *P < 0.05, repeated three times. **D** Transwell analysis of BC cell lines MDA-MB-231 and MCF7 treated with miR-338-3p mimic. Left: Representative image of the Transwell experiment; Right: Statistical analysis of the Transwell experiment, **P < 0.01, ***P < 0.001, repeated three times. **E** Colony formation experiments in BC cell lines MDA-MB-231 and MCF7 treated with miR-338-3p mimic. Left: Representative image of the colony formation experiment; Right: Statistical analysis of the colony formation experiment. ***P < 0.001, ****P < 0.0001, repeated three times. **F** Transwell analysis after BC cells MDA-MB-231 and MCF7 overexpressed circGSK3β and miR-338-3p. Left: Representative image of the Transwell experiment; Right: Statistical analysis of the Transwell experiment. *P < 0.05, **P < 0.01, repeated three times. **G** Colony formation experiments after overexpressing circGSK3β in BC cells MDA-MB-231 and MCF7, and overexpressing circGSK3β and miR-338-3p. Left: Representative image of the colony formation experiment; Right: Statistical analysis of the colony formation experiment, ****P < 0.0001, repeated three times. Ctrl means scrambled RNA control.

Existing studies have shown that circRNAs can influence tumor occurrence and development through various mechanisms, such as acting as miRNA sponges, binding to RBPs, acting as transcription factors, or being translated into proteins [8, 31, 32]. Our findings have revealed that circGSK3β is predominantly expressed in the cytoplasm. Therefore, we hypothesized whether circGSK3β functions as a miRNA sponge in BC. To investigate this, we predicted potential downstream miRNAs that could interact with circGSK3β using the Starbase and Circinteractom databases. The bioinformatics analysis identified hsa-miR-338-3p as the only common miRNA that could potentially interact with circGSK3β (Fig. 3A). Additionally, we observed that overexpression of circGSK3β led to reduced expression of miR-338-3p in MDA-MB-231 cells (Fig. 3B). To verify the binding between circGSK3β and miR-338-3p, we first predicted their binding sites through the Circinteractom database (Supplementary Fig. S2A). Subsequently, we constructed luciferase reporter gene vectors containing circGSK3β mutant and wild-type sequences based on their interaction sites (Supplementary Fig. S2B). Furthermore, we designed a mimic of miR-338-3p and confirmed its overexpression efficiency in MDA-MB-231 and MCF7 cells using qRT-PCR (Supplementary Fig. S2C). We then conducted a dual-luciferase reporter assay to validate the interaction between circGSK3β and miR-338-3p. The results demonstrated that the miR-338-3p mimic significantly reduced the luciferase activity of wild-type circGSK3β, while no significant change was observed for mut-circGSK3β (Fig. 3C, Supplementary Fig. S2D). The results of matrigel invasion and scratch wound healing assays revealed that overexpression of circGSK3β enhanced BC cells invasion and migration. Compared with overexpression of circGSK3β, overexpression of mut-circGSK3β inhibited the invasion and migration of BC cells (Supplementary Fig. S3A–D). Similarly, colony formation experiments demonstrated that overexpression of circGSK3β enhanced BC cell proliferation. Compared with overexpression of circGSK3β, overexpression of mut-circGSK3β inhibited the proliferation of BC cells (Supplementary Fig. S3E–F). In summary, our findings suggest that circGSK3β may function as a molecular sponge for miR-338-3p in BC, although its precise role in BC cells requires further elucidation.

To ascertain whether circGSK3β‘s tumor-promoting effect is mediated through its sponging of miR-338-3p, we first examined the role of miR-338-3p in BC progression. We assessed the expression level of miR-338-3p in immortalized breast epithelial cells (MCF-10A) and BC cell lines (ZR75-1, MX-1, HS578T, MDA-MB-468, MDA-MB-231) through qRT-PCR experiments. The results revealed a downward trend in miR-338-3p expression levels in BC cell lines compared to normal breast epithelial cells MCF-10A (Supplementary Fig. S4A). Furthermore, Kaplan-Meier Plotter analysis on the online platform demonstrated that patients with lower miR-338-3p expression had a significantly worse overall survival rate (P = 0.00021) (Supplementary Fig. S4B). Subsequently, matrigel invasion, scratch wound healing, and colony formation assays showed that overexpression of miR-338-3p in MDA-MB-231 and MCF7 cell lines inhibited BC cell invasion, migration, and proliferation (Fig. 3D, E, Supplementary Fig. S4C). To elucidate whether circGSK3β‘s tumor-promoting effect occurs via its sponging of miR-338-3p, we conducted rescue experiments in MDA-MB-231 and MCF7 cell lines. The results of matrigel invasion and scratch wound healing assays revealed that overexpression of circGSK3β significantly enhanced BC cell invasion and migration. However, this tumor-promoting effect induced by circGSK3β was abrogated upon introduction of miR-338-3p (Fig. 3F, Supplementary Fig. S4D, E). Similarly, colony formation experiments demonstrated that overexpression of circGSK3β in MCF7 cells significantly enhanced BC cell proliferation. Notably, this tumor-promoting effect induced by circGSK3β was also nullified upon introduction of miR-338-3p (Fig. 3G). Furthermore, matrigel invasion and scratch wound healing assays confirm that wild-type circGSK3β can exert its function through miR338-3p, while the mutant circGSK3β significantly weakens this effect (Supplementary Figures S5A–D). The colony formation experiments yielded similar results (Supplementary Fig. S5E, F). In conclusion, circGSK3β acts as a sponge for miR-338-3p in breast cancer and promotes breast cancer progression.

### CircGSK3β upregulates PDL1 expression through miR-338-3p/PRMT5

**Fig. 4:**
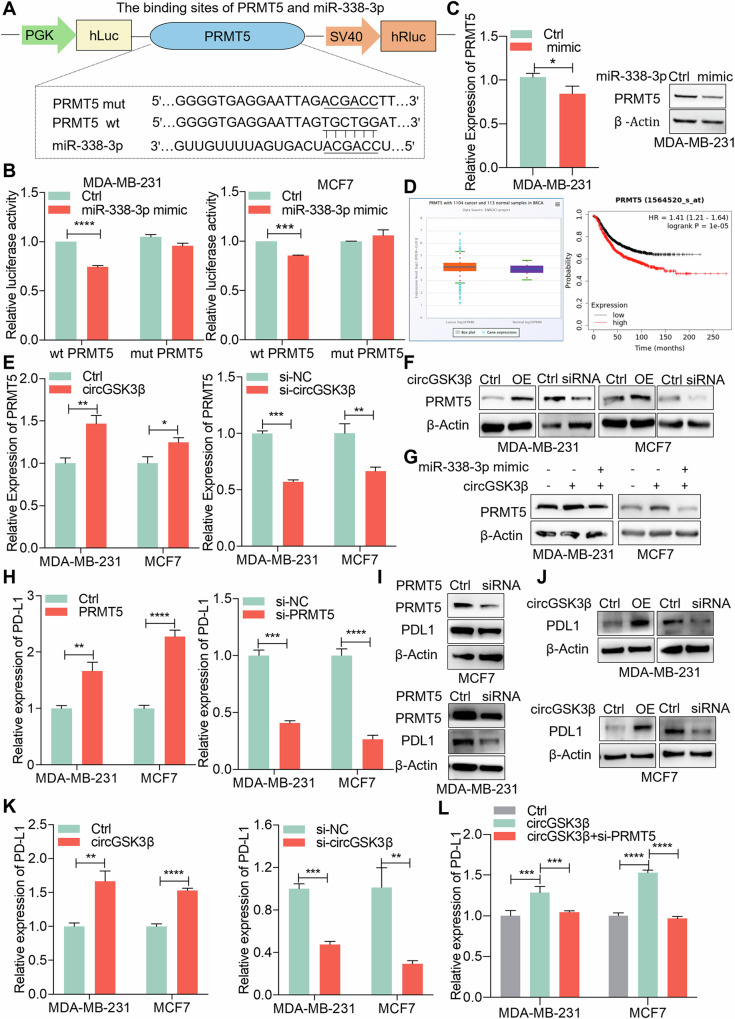
CircGSK3β upregulates PDL1 expression through miR-338-3p/PRMT5. **A** Schematic diagram of constructing PRMT5 mutant and wild-type luciferase reporter gene vectors. **B** Luciferase reporter gene assay after co-transfection of miR-338-3p mimic and PRMT5 3’UTR wild-type or mutant luciferase reporter gene vector into MDA-MB-231 and MCF7 cells. wt: wild type, mut: mutant type, ***P < 0.001, ****P < 0.0001. **C** Detection of the effect of miR-338-3p on PRMT5 expression. Left: qRT-PCR experiment to detect the mRNA expression level of PRMT5 after overexpressing miR-338-3p. Right: Western Blot experiment to detect the PRMT5 protein expression level after transfection with miR-338-3p mimic, *P < 0.05. **D** Analysis of PRMT5 expression in BC tissues and adjacent tissues using the ENROCI database (left), and the relationship between PRMT5 expression and prognosis of BC patients using Kaplan-Meier Plotter (right), *P* < 0.01. **E** qRT-PCR detecting the mRNA expression level of PRMT5 after overexpression/knockdown of circGSK3β, *P < 0.05, **P < 0.01, ***P < 0.001. **F** Western blot detecting PRMT5 protein expression level after overexpression/knockdown of circGSK3β. **G** Western blot detecting PRMT5 protein expression levels in cells overexpressing circGSK3β and overexpressing circGSK3β while overexpressing miR-338-3p. **H** qRT-PCR detecting the mRNA expression level of PDL1 after overexpression/knockdown of PRMT5. *P < 0.05, **P < 0.01, ***P < 0.001. **I** Western blot detecting the expression level of PDL1 protein after PRMT5 knockdown in BC cells MDA-MB-231 and MCF7. **J** Western blot detecting the expression level of PDL1 protein after circGSK3β overexpression/knockdown in BC cells MDA-MB-231 and MCF7. **K** qRT-PCR detecting the mRNA expression level of PDL1 after overexpression/knockdown of circGSK3β in BC cells MDA-MB-231 and MCF7. **L** qRT-PCR detecting the mRNA expression level of PDL1 after overexpressing circGSK3β and overexpressing circGSK3β while knocking down PRMT5 in BC cells MDA-MB-231 and MCF7.

To delve deeper into the potential role and mechanism of circGSK3β/miR-338-3p in BC immune regulation, we utilized miRPathDB and miRWalk3 for bioinformatics analysis to predict the downstream targets of miR-338-3p [33–36]. The results suggested that PRMT5 might be a potential downstream target of miR-338-3p. To ascertain whether miR-338-3p binds to the 3’UTR of PRMT5 mRNA, we initially constructed mutant and wild-type luciferase reporter vectors of PRMT5 based on the putative binding sites of PRMT5 and miR-338-3p (Fig. 4A). Results from the dual-luciferase reporter gene assay showed that the miR-338-3p mimic significantly attenuated the luciferase activity of wild-type PRMT5, while no difference was observed for mut-PRMT5 (Fig. 4B). Subsequently, we delved into the regulatory effect of miR-338-3p on PRMT5 expression in BC cells. qRT-PCR results demonstrated that overexpression of miR-338-3p reduced the mRNA level of PRMT5 in MDA-MB-231 cells. Concurrently, Western blot analysis showed that overexpression of miR-338-3p significantly decreased the protein level of PRMT5 (Fig. 4C). Furthermore, according to analysis from the ENROCI database, PRMT5 is significantly upregulated in BC tissues. Additionally, analysis from the Kaplan-Meier Plotter website revealed that BC patients with high PRMT5 expression exhibit a poor prognosis (*p* < 0.01) (Fig. 4D). To elucidate whether circGSK3β regulates the expression of the downstream target gene PRMT5, our qRT-PCR and Western blot results confirmed that circGSK3β positively regulates PRMT5 expression in BC cells (Fig. 4E, F). Moreover, Western blot analysis also demonstrated that miR-338-3p reversed the circGSK3β-induced upregulation of PRMT5 in MDA-MB-231 and MCF7 cell lines (Fig. 4G). Taken together, these findings confirm that circGSK3β upregulates the expression of PRMT5 through sponge-mediated regulation of miR-338-3p.

Previous research findings have indicated that protein arginine methyltransferase 5 (PRMT5) is closely linked to tumor occurrence and belongs to the type II protein arginine methyltransferase family [18]. PRMT5 can either activate or repress gene expression by generating histone marks such as H3R2me2s, H3R8me2s, or H4R3me2s [20, 37–40]. PRMT5 has been reported to impact the anti-tumor function of PD-L1 by regulating its expression, thereby promoting cancer progression [25]. Given that circGSK3β upregulates PRMT5 expression by sponging miR-338-3p, we aimed to further ascertain whether circGSK3β could influence PD-L1 expression by regulating PRMT5. To investigate this, we initially examined the effect of PRMT5 on PD-L1 expression in breast cancer MDA-MB-231 and MCF7 cell lines. The qRT-PCR results revealed that overexpression of PRMT5 promoted PD-L1 mRNA levels, whereas knockdown of PRMT5 inhibited PD-L1 mRNA levels (Fig. 4H). Correspondingly, Western blot results demonstrated that knocking down PRMT5 suppressed the protein expression level of PD-L1 (Fig. 4I). Additionally, overexpression of circGSK3β in breast cancer MDA-MB-231 and MCF7 cell lines enhanced the protein expression and mRNA levels of PD-L1, whereas knocking down circGSK3β attenuated the protein expression and mRNA levels of PD-L1 (Fig. 4J, K). Finally, we conducted rescue experiments in breast cancer MDA-MB-231 and MCF7 cell lines. Knocking down PRMT5 reversed the effect of circGSK3β on PD-L1 expression (Fig. 4L). In summary, our results suggest that circGSK3β can upregulate PD-L1 expression through the miR-338-3p/PRMT5 pathway.

### PRMT5 regulates PDL1 expression through histone H3K4 trimethylation (H3K4me3)

**Fig. 5:**
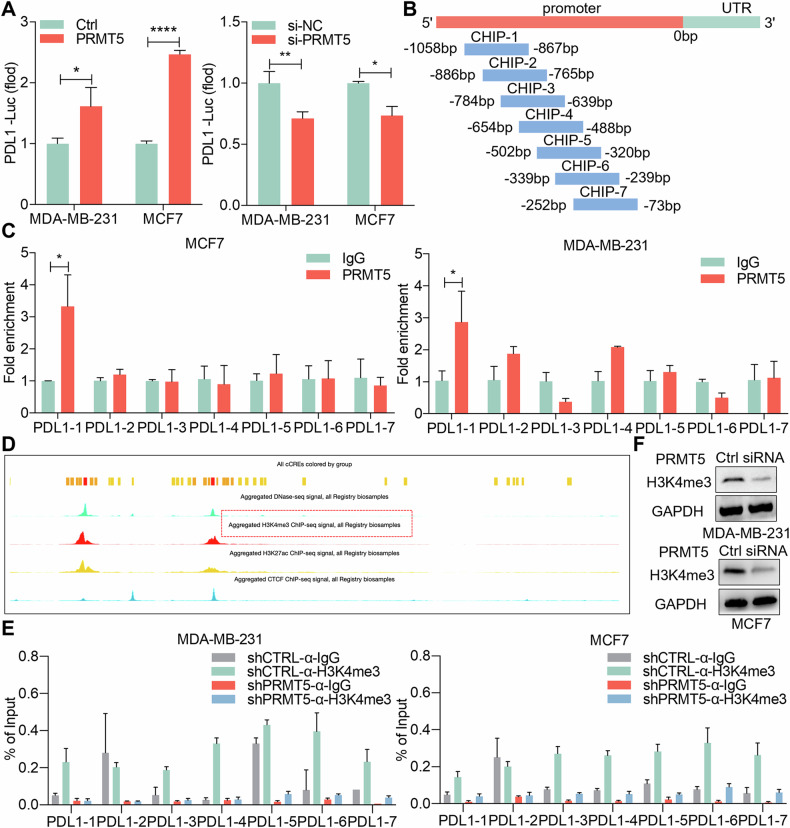
PRMT5 regulates PDL1 expression through histone H3K4 trimethylation (H3K4me3). **A** Luciferase reporter gene assay using luciferase reporter gene vectors overexpressing/knocking down PRMT5 and PDL1 in MDA-MB-231 and MCF7 cells. *P < 0.05, **P < 0.01, ***P < 0.0001. **B** Schematic diagram of primer design scheme for PDL1 promoter and its fragments in ChIP detection. **C** Detection of PRMT5 or IgG enrichment at the PDL1 promoter by ChIP PCR. **D** Analysis of H3K4me3 marks enrichment in the human proximal cd274 promoter region using the ENCODE website. **E** ChIP PCR detecting the enrichment of PRMT5, H3K4me3, or IgG at the PDL1 promoter. **F** Western blot detecting the expression level of H3K4me3 protein in breast cancer cells MDA-MB-231 and MCF7 after PRMT5 knockdown.

Our investigation aimed to determine whether PRMT5, a known gene expression regulator, directly regulates PD-L1 transcription following our confirmation that circGSK3β upregulates PD-L1 expression via miR-338-3p/PRMT5. Using a PD-L1 promoter plasmid and luciferase reporter gene experiment, we confirmed PRMT5’s role in promoting PD-L1 transcription (Fig. 5A). To further confirm whether PRMT5 directly binds to the promoter region of PD-L1, we designed ChIP PCR primers for the PD-L1 promoter region (Fig. 5B). ChIP PCR assays demonstrated PRMT5’s direct binding to the PD-L1 promoter region (Fig. 5C). While PRMT5 typically modulates gene expression via symmetrical methylation of histone arginine residues, previous studies suggested it couldn’t influence PD-L1 transcription through this mechanism [25]. PRMT5 methylates H3R2 (H3R2me2s), recognized by WDR5, enabling recruitment of the SET1/MLL complex and histone H3K4 trimethylation (H3K4me3) [23]. However, it remained unexplored whether PRMT5 regulates PD-L1 expression via H3K4me3. Our speculation was supported by the enrichment of H3K4me3 marks at the PD-L1 promoter in a PRMT5-dependent manner in BC cell lines, as revealed by bioinformatics and ChIP analyses (Fig. 5D, E). Furthermore, PRMT5 depletion altered the overall level of H3K4me3 (Fig. 5F). Thus, our findings suggest that PRMT5 recruits to the PD-L1 promoter and mediates PD-L1 expression through H3K4me3, thereby influencing tumor immunity in BC.

### CircGSK3β promotes immune evasion and tumor progression through miR-338-3p/PRMT5

**Fig. 6:**
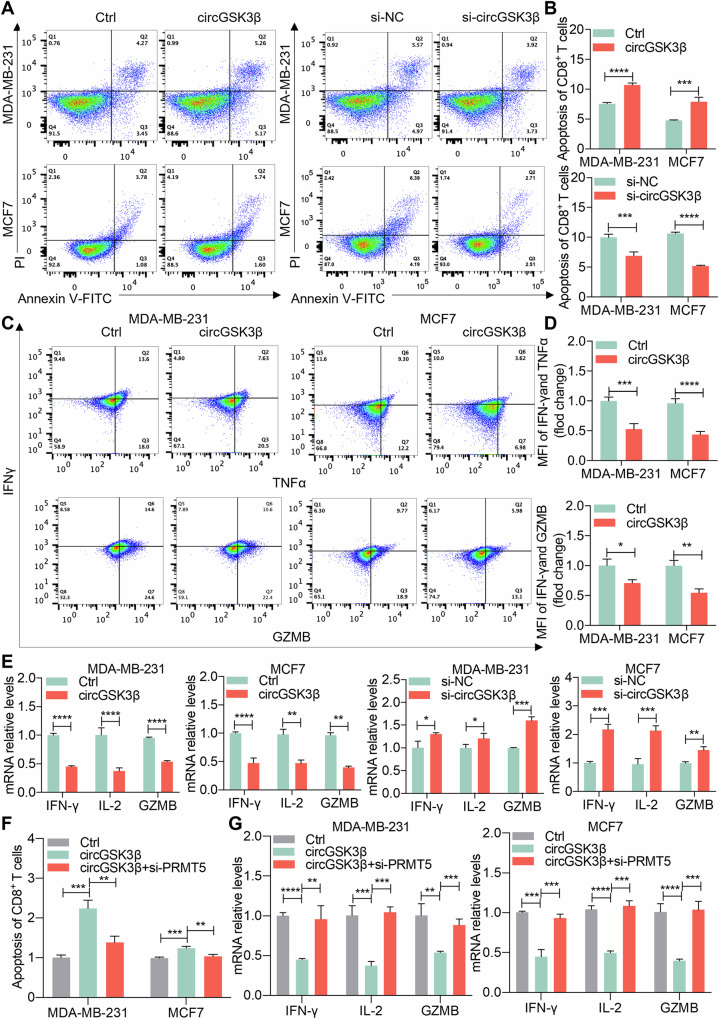
CircGSK3β promotes immune evasion and tumor progression through miR-338-3p/PRMT5. **A**, **B** BC cells MDA-MB-231 and MCF7 knocking down circGSK3β were co-cultured with T cells. Analysis of primary T cell apoptosis by flow cytometry. **B** Counting of early apoptotic cells (Annexin V + , PI-) and late apoptotic cells (Annexin V + , PI + ) as the proportion of apoptotic T cells. ***P < 0.001, ****P < 0.0001, repeated three times. **C**, **D** BC cells MDA-MB-231 and MCF7 co-cultured with T cells after overexpressing circGSK3β and overexpressing circGSK3β while knocking down PRMT5. Analysis of secretion levels of IFN-γ, TNF-α, and GZMB in primary T cells by flow cytometry. **D** Counting the proportion of secretion levels of IFN-γ, TNF-α, and GZMB in each group, *P < 0.05, **P < 0.01, ***P < 0.001, ****P < 0.0001, repeated three times. **E** qRT-PCR identifying IFN-γ, IL-2, and GZMB mRNA levels in primary T cells co-cultured with BC cells MDA-MB-231 and MCF7. *P < 0.05, **P < 0.01, ***P < 0.001, ****P < 0.0001, repeated three times. **F** BC cells MDA-MB-231 and MCF7 co-cultured with T cells after overexpressing circGSK3β and overexpressing circGSK3β while knocking down PRMT5. Analysis of primary T cell apoptosis by flow cytometry, and counting the proportion of apoptotic T cells. **P < 0.01, **P < 0.001, repeated three times. **G** BC cells MDA-MB-231 and MCF7 co-cultured with T cells after overexpressing circGSK3β and overexpressing circGSK3β while knocking down PRMT5. Identification of IFN-γ, IL-2, and GZMB mRNA levels in primary T cells by qRT-PCR. **P < 0.01, ***P < 0.001, ****P < 0.0001, repeated three times.

Our investigation confirmed that circGSK3β can recruit H3K4me3 through PRMT5 to enhance PD-L1 expression. To verify that circGSK3β promotes immune evasion and tumor progression via miR-338-3p/PRMT5, we first assessed its impact on immune evasion in BC cells. Co-culturing activated human primary T cells with BC cells overexpressing or knocking down circGSK3β revealed that circGSK3β overexpression in MDA-MB-231 and MCF7 cells promoted T cell apoptosis, while circGSK3β knockdown inhibited T cell apoptosis (Fig. 6A, B). This suggests that circGSK3β hinders the cytotoxic function of T cells against tumor cells. T cells typically secrete IFN-γ and other cytokines to eliminate tumor cells. Flow cytometry results indicated that circGSK3β overexpression reduced IFN-γ, TNFα, and GZMB secretion levels in T cells co-cultured with MDA-MB-231 and MCF7 cells, while circGSK3β knockdown increased these levels (Fig. 6C, D; Supplementary fig. S6A, B). Moreover, qRT-PCR revealed that circGSK3β overexpression decreased mRNA expression levels of IFN-γ, IL-2, and GZMB in co-cultured T cells, while circGSK3β knockdown elevated their expression (Fig. 6E). Thus, circGSK3β effectively suppresses the production of IFN-γ and other cytokines by T cells surrounding tumor cells, thereby dampening their cytotoxic effects. Subsequently, we investigated whether circGSK3β affects BC immune evasion through PRMT5. Knocking down PRMT5 in MDA-MB-231 and MCF7 cells reversed circGSK3β‘s promotion of T cell apoptosis (Fig. 6F; Supplementary fig. S6C). qRT-PCR results demonstrated that PRMT5 knockdown reversed the inhibition of T cell factor secretion (IFN-γ, IL-2, and GZMB) induced by circGSK3β (Fig. 6G). ELISA analysis further revealed that PRMT5 knockdown counteracted the inhibition of IFN-γ secretion by circGSK3β (Supplementary fig. S6D). In summary, our findings indicate that circGSK3β regulates PD-L1 expression in vitro and dampens cytotoxic T cell activity through PRMT5, thereby mediating BC cell immune evasion and tumor progression.

## Discussion

Our study underscores the significance of circGSK3β in BCprogression. Being a novel type of non-coding RNA, circGSK3β emerges as a highly conserved covalent closed-loop RNA with a reverse 5’ to 3’ end head-to-tail connection. While traditional molecular techniques may not readily apply due to circRNA’s lack of 3’ and 5’ free ends, recent advances in polyadenylated enrichment and RNA-seq techniques have enabled extensive circRNA exploration in cancer research [41–44]. Indeed, various circRNAs have been implicated in cancer, including breast cancer, liver cancer and papillary thyroid cancer, where they exert their effects via diverse mechanisms such as acting as competing endogenous RNAs (ceRNAs) or regulating downstream target proteins [45–53]. However, the role of circGSK3β, a novel oncogenic circRNA derived from the GSK3β gene, in BC remains unexplored. Our study fills this gap by demonstrating that circGSK3β is highly expressed in BC cells and plays a pivotal role in promoting BC progression, making it a potential diagnostic, prognostic marker, and therapeutic target for BC.

The molecular mechanisms underlying circRNA involvement in tumors are multifaceted. One common mechanism involves circRNAs acting as miRNA sponges [54–56]. Given its cytoplasmic localization, we hypothesized that circGSK3β might interact with miRNAs. Through bioinformatics analysis, we identified hsa-miR-338-3p as a potential interaction partner of circGSK3β. Subsequent dual-luciferase reporter assays confirmed the tight binding between circGSK3β and hsa-miR-338-3p. Further investigations revealed that circGSK3β promotes BC cell proliferation, migration, and invasion through hsa-miR-338-3p, shedding light on the tumor-suppressive role of hsa-miR-338-3p in BC. Additionally, bioinformatics analysis implicated PRMT5 as a potential downstream target of hsa-miR-338-3p. Consistent with this, dual-luciferase reporter gene and qRT-PCR assays confirmed PRMT5 as a downstream target of hsa-miR-338-3p. Moreover, we discovered that circGSK3β upregulates PRMT5 expression by sponging miR-338-3p, highlighting a novel mechanism by which circRNAs regulate downstream target genes.

PRMT5, a type II enzyme, modulates gene expression by catalyzing symmetric dimethylarginine from histones and non-histone proteins [57, 58]. Through histone modification, PRMT5 can either drive or inhibit gene expression [59, 60]. Notably, PRMT5 has been implicated in cancer development and immuno-oncology, where it affects the anti-tumor function of PD-L1 by regulating its expression [24, 60–62]. Building on this knowledge, we investigated whether circGSK3β influences tumor immunity by modulating PD-L1 expression through PRMT5. Our study revealed that circGSK3β promotes PD-L1 expression, and knockdown of PRMT5 reversed this effect, indicating that circGSK3β upregulates PD-L1 expression through miR-338-3p/PRMT5. Moreover, we found that PRMT5 directly binds to the PD-L1 promoter, thereby promoting PD-L1 transcription, likely via H3K4me3 modification. These findings provide new insights into the regulatory mechanisms of circGSK3β in BC immune escape and tumor progression.

One limitation of this study is that due to the limitations of available specimens, the correlation between CircGSK3β and the prognosis of BC patients cannot be verified. Our research group will further strengthen the collection of relevant specimens. In addition, this study only detected the effect of CircGSK3β on CD8^+^ T cells in vitro, and animal experiments will be needed for further verification in the future.

**Fig. 7:**
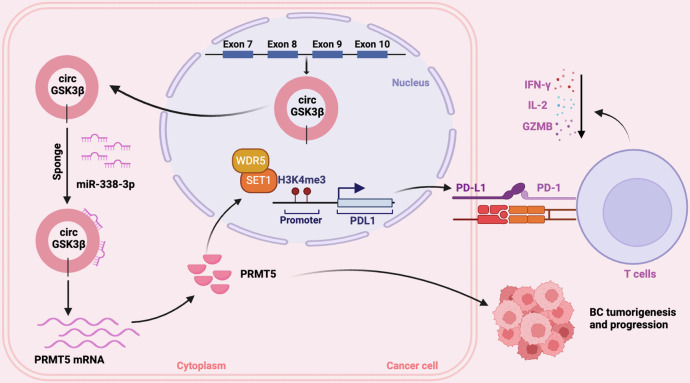
Working model of CircGSK3β promoting breast cancer progression and immune evasion through the miR-338-3p-PRMT5-H3K4me3 axis. Schematic representation illustrating the involvement of the circGSK3β-miR-338-3p-PRMT5-H3K4me3 axis in promoting breast cancer progression and immune evasion (created with BioRender.com).

In conclusion, our study elucidates the pivotal role of circGSK3β in BC progression. Through its interaction with miR-338-3p and subsequent regulation of PRMT5 and PD-L1, circGSK3β promotes BC cell proliferation, migration, invasion, and immune evasion. These findings position circGSK3β as a promising diagnostic, prognostic biomarker, and therapeutic target for BC. Moreover, our study sheds light on the complex interplay between circRNAs, miRNAs, and downstream target genes in BC progression, offering new avenues for understanding and combating this disease (Fig. 7).

## Materials methods

### Cell lines and cell culture

Cell lines including MDA-MB-231, MCF7, MDA-MB-468, HST578T, ZR75-1, MX-1, and MCF-10A were obtained from our laboratory and cultured under specific conditions. MDA-MB-231, MCF7, MDA-MB-468, HST578T, ZR75-1, and MX-1 breast cancer cell lines were maintained in DMEM medium supplemented with 10% fetal bovine serum (Gibco Life Technologies, NY, USA), penicillin (100 U/mL), and streptomycin (100 μg/mL) at 5% CO_2_ and 37 °C. Human mammary epithelial MCF-10A cells were cultured in DMEM medium containing 5% horse serum, epidermal growth factor, insulin, and hydrocortisone, also at 5% CO_2_ and 37 °C. Regular mycoplasma testing was conducted on all cell lines to ensure quality control.

### Vectors, siRNA sequences, and cell transfection

The PCR-amplified product of human circGSK3β was inserted into the pcDNA3.1 vector to generate an expression vector carrying circGSK3β, which was subsequently confirmed by sequencing. Additional recombinant plasmids included pcDNA3.1(+), pcDNA3.1-HA-PRMT5, and a luciferase reporter plasmid corresponding to the mutation, which were constructed by Changsha Youbao Biotechnology Co., Ltd. SiRNAs targeting circGSK3β, PRMT5, and control siRNAs, as well as miR-338-3p mimics, were procured from RiboBio. Transfections of siRNAs and gene plasmids were performed using the riboFECT CP Transfection Kit (RIBOBIO Co., China) or Neofect DNA Transfection Reagent (Neofect Biotech, China) following the manufacturer’s instructions. Detailed sequences of the siRNAs and miRNA mimics utilized in this study are provided in supplementary table S1.

### Quantitative real-time polymerase chain reaction (qRT-PCR)

Total RNA was extracted from human BC cell lines using TRIzol reagent (Invitrogen, MA, USA) following the manufacturer’s protocol. Reverse transcription of mRNA and miRNA was carried out using random primers and stem-loop primers, respectively. Quantitative real-time PCR (qRT-PCR) was conducted on a Bio-Rad CFX96 qPCR system using the SYBR Green PCR Master Mix (Vazyme, Nanjing, China) kit. Fold changes were calculated using the 2-ΔΔCT method for relative quantification. The isolation of nuclear and cytoplasmic fractions from cells was performed using the Cytoplasmic & Nuclear RNA Purification Kit (NORGEN BIOTEK, Canada) according to the manufacturer’s instructions, and RNA was extracted from both fractions. Subsequently, qRT-PCR was conducted to assess the expression ratio of specific RNA molecules between the nuclear and cytoplasmic fractions. GAPDH and U6 were utilized as cytoplasmic and nuclear markers, respectively. Detailed primer sequences are provided in supplementary table S2.

### Western blot analysis

Western blot analysis was conducted following previously described methods [63]. In brief, proteins were extracted from BC cell lines, and the protein concentration was determined using the BCA method protein quantification kit (Thermo Scientific, Rockford, IL, USA). The antibodies utilized are listed in supplementary table S3.

### Colony formation, migration and invasion assays

The colony formation assay was conducted to evaluate cell proliferation ability, while the Transwell assay was employed to assess cell invasion ability. Additionally, wound healing assays were performed to evaluate cell migration ability. Detailed protocols for these assays can be found in our previous study [64].

### Dual luciferase reporter gene assay

The sequences of circGSK3β and PRMT5, along with their corresponding mutations, were designed, synthesized, and inserted into luciferase reporter vectors, resulting in constructs termed circGSK3β-WT, circGSK3β-Mut, PRMT5-WT, and PRMT5-Mut. These constructs were then co-transfected with either miR-338-3p mimic or control mimic into MDA-MB-231 and MCF7 cells, respectively. Subsequently, the relative luciferase activity was assessed using a dual-luciferase assay kit (Promega, USA) following the manufacturer’s instructions.

### Chromatin immunoprecipitation (ChIP) assay

ChIP analysis was conducted following the manufacturer’s instructions with the ChIP Assay kit (Beyotime Biotechnology, Shanghai, China). Briefly, BC cells were cross-linked using 37% formaldehyde for 10 min at room temperature, followed by sonication to shear the chromatin. Immunoprecipitation of chromatin-protein complexes was carried out using anti-PRMT5 (1:100, 79998, CST) and anti-H3K4me3 (1:50, DF6935, Affinity Biosciences), or IgG control. The resulting DNA fragments were then utilized as templates for qRT-PCR based on specific primers (supplementary table S4).

### Isolation, preparation and expansion of human CD8^+^ T cells

Peripheral blood mononuclear cells (PBMCs) were isolated from peripheral blood obtained from healthy donors using the Ficoll-Hypaque method (Cytiva, USA) as per the manufacturer’s instructions. T cells were then expanded in vitro by supplementing PBMCs with CD3/CD28 MACSiBeads (Miltenyi, Germany), along with 15 ng/mL IL-2, 5 ng/mL IL-7, and 10 ng/mL IL-15 (Sino Biological), and incubating for 8 days. Throughout the experiment, fresh culture medium and cytokines were replenished every 2 days.

### Flow cytometry

Transfected tumor cells and activated human primary T cells were co-cultured at a ratio of 1:10 for 3 h. T cells were labeled using fluorescently labeled CD3 and CD8 antibodies. T cell apoptosis was detected using the apoptosis detection kit (AP101, Lianke Biotech) and flow cytometry, following the manufacturer’s instructions. This kit stains Annexin V and PI, allowing for the identification of early apoptotic cells (Annexin V + , PI-) and late apoptotic cells (Annexin V + , PI + ). Additionally, a Golgi transport blocker, 25 ng/mL BFA (Selleck), was added. After 3 h of co-culture, T cells were collected and stained with cell membrane antibodies CD3 and CD8. Following pretreatment with a membrane-breaking agent, fluorescently labeled cytokine antibodies for IFN-γ, TNFα, and GZMB were added for incubation. The levels of IFN-γ, TNFα, and GZMB in T cells were then detected using a DxPAthenaTM flow cytometer (Cytek, USA). Data analysis was performed using FlowJo v10 software (Treestar).

### Statistical analysis

ImageJ was employed to quantify the overall area in the scratch experiment and the number of tumor cells that penetrated the basement membrane in the Transwell assay. Statistical analyses were conducted using GraphPad Prism 8 to generate quantitative histograms. Data were presented as mean ± standard deviation, and t-tests were utilized to compare data between groups. All experiments were performed with triplicate samples. Survival analysis was conducted using Kaplan-Meier Plotter and log-rank test. Statistical significance was defined as *p* < 0.05.

## Supplementary information

Supplementary Materials

wb image

## Data Availability

The data that support the findings of this study are available from the corresponding author upon reasonable request.
