# Supplementary material for: CircGSK3β mediates PD-L1 transcription through miR-338-3p/PRMT5/H3K4me3 to promote breast cancer cell immune evasion and tumor progression
Source: Cell Death Discov. 2024 Oct 4;10:426. doi: 10.1038/s41420-024-02197-8 (PMC11452702; doi:10.1038/s41420-024-02197-8)
Supplement: Supplementary file 1 — Supplementary Materials [file 41420_2024_2197_MOESM1_ESM.docx]

**Supplementary Figure 1**

**
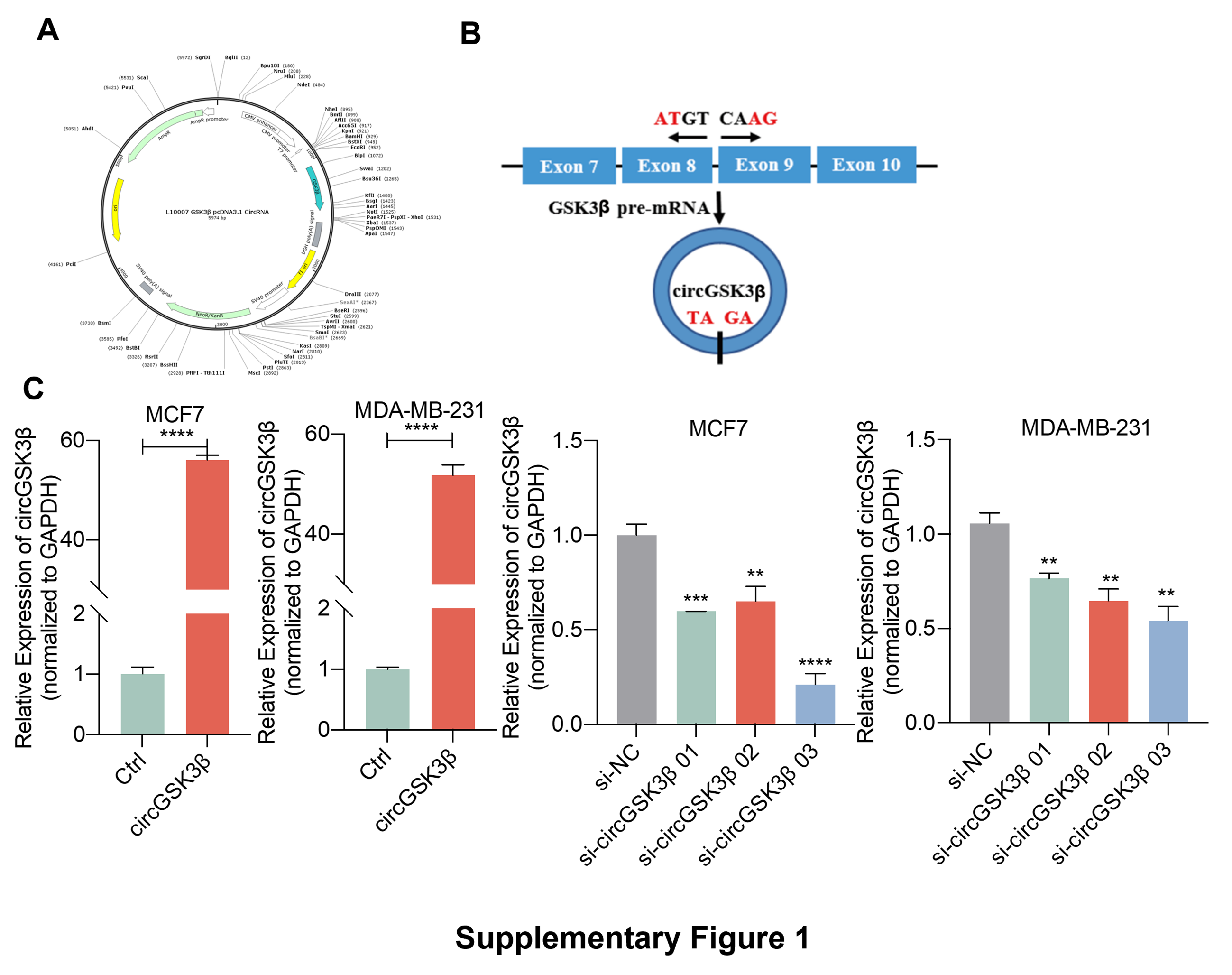
**

**Supplementary Figure 1. CircGSK3β promotes breast cancer progression (construction of circGSK3β plasmid and siRNAs).** (A) Schematic diagram depicting the construction of L10007 GSK3β pcDNA 3.1 CircRNA plasmid. (B) Illustration of the design targets for siRNA. (C) RT-PCR experiment evaluating the transient transfection efficiency of circGSK3β plasmid and the knockdown effect of circGSK3β. Statistical significance indicated as ***P* < 0.01, *****P* < 0.0001. The experiment was repeated three times.

**Supplementary Figure 2**

**
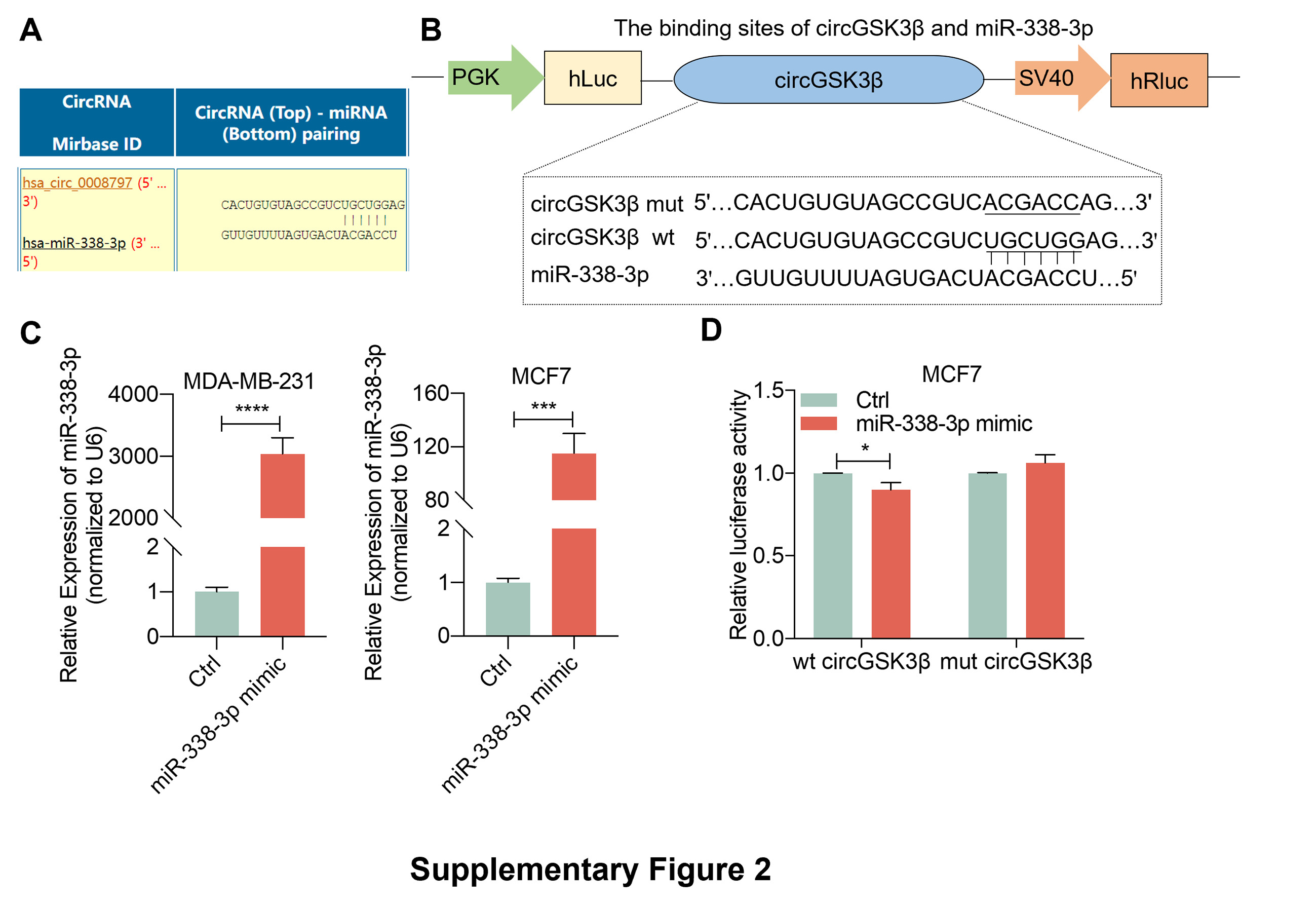
**

**Supplementary Figure 2. CircGSK3β acts as a sponge for miR-338-3p in breast cancer.** (A) Prediction of the binding site of circGSK3β on miR-338-3p using Circinteractom. (B) Schematic representation of constructing circGSK3β mutant and wild-type luciferase reporter gene vectors. (C) RT-PCR experiment assessing the overexpression efficiency of miR-338-3p mimic in breast cancer cell lines MDA-MB-231 and MCF7. Statistical significance indicated as ****P* < 0.001, *****P* < 0.0001. The experiment was repeated three times. (D) Luciferase reporter gene assay of miR-338-3p mimics and circGSK3β wild-type or mutant luciferase reporter gene co-transfected into MCF7 cells. Statistical significance indicated as **P* < 0.05. The experiment was repeated three times.

**Supplementary Figure 3**

**
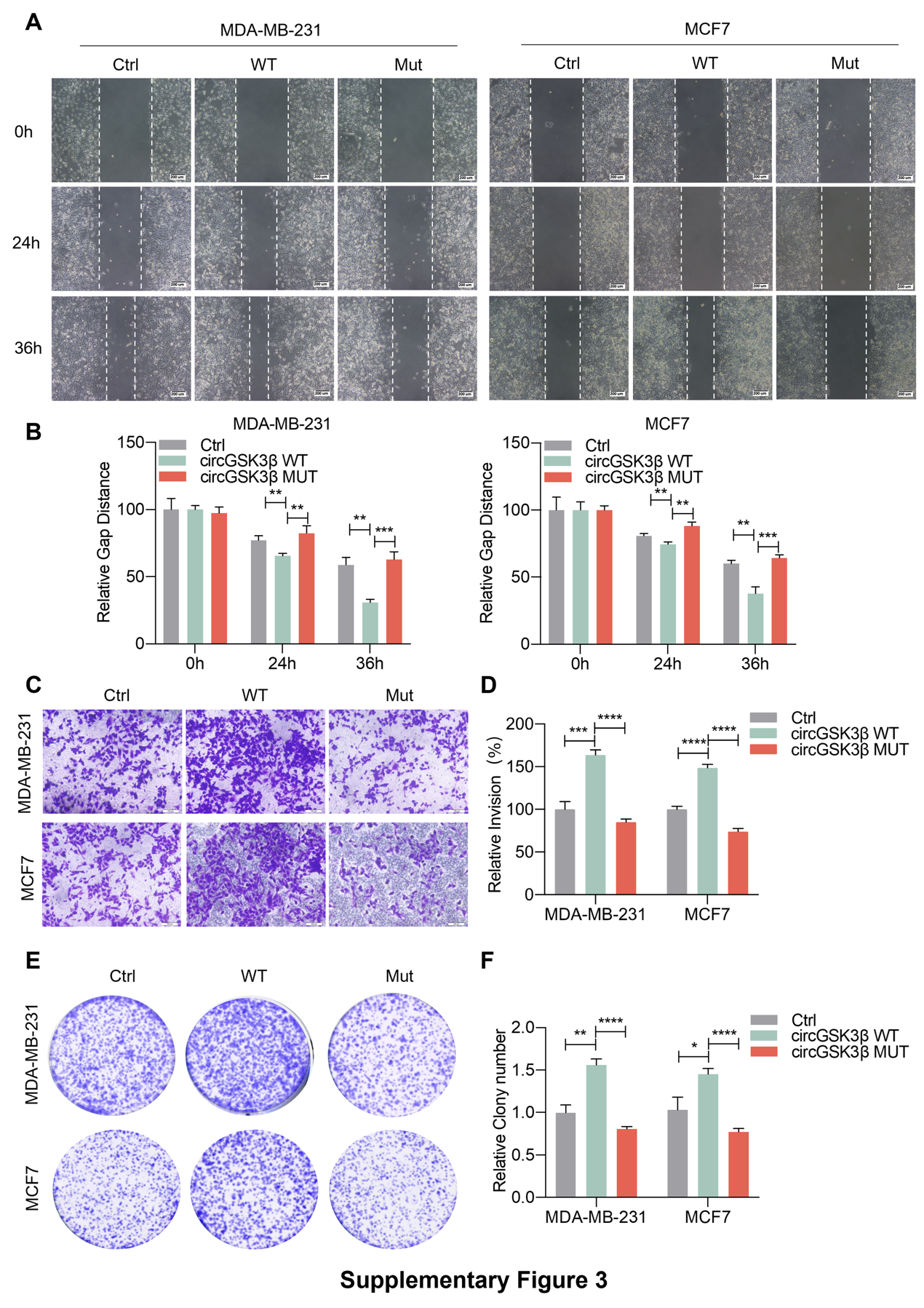
**

**Supplementary Figure 3. Effects of wild-type and mutant CircGSK3β on invasion, migration and proliferation of breast cancer cells.** (A, B) Wound-healing experiments conducted in breast cancer cell lines MDA-MB-231 and MCF7 transfected with empty vector (Ctrl), overexpressing circGSK3β wild-type (WT), or circGSK3β mutant (Mut). (A) Representative image of the wound-healing experiment; (B) Statistical analysis of the wound-healing experimen. Statistical significance indicated as ***P* < 0.01, ****P* < 0.001. The experiment was repeated three times. (C, D) Transwell analysis of BC cell lines MDA-MB-231 and MCF7 transfected with empty vector (Ctrl), overexpressing circGSK3β wild-type (WT), or circGSK3β mutant (Mut). (C) Representative image of the Transwell experiment; (D) Statistical analysis of the Transwell experiment. Statistical significance indicated as ****P* < 0.001, *****P* < 0.0001. The experiment was repeated three times. (E, F) Colony formation experiments in BC cell lines MDA-MB-231 and MCF7 transfected with empty vector (Ctrl), overexpressing circGSK3β wild-type (WT), or circGSK3β mutant (Mut). (E) Representative image of the colony formation experiment; (F) Statistical analysis of the colony formation experiment. Statistical significance indicated as **P* < 0.05, ***P* < 0.01, *****P* < 0.0001. The experiment was repeated three times.

**Supplementary Figure 4**


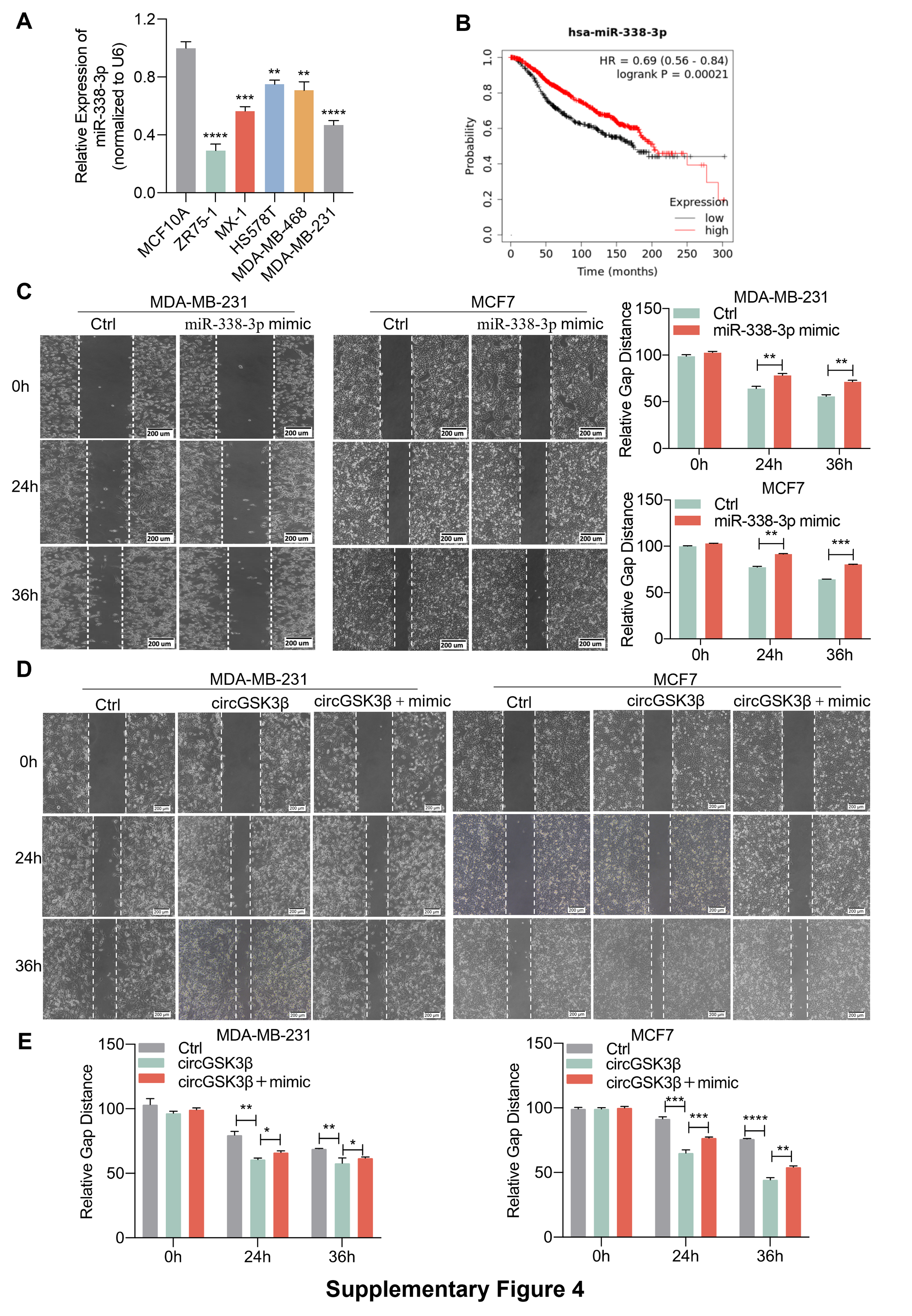


**Supplementary Figure 4. CircGSK3β acts as a sponge for miR-338-3p in breast cancer and promotes breast cancer progression** .(A) RT-PCR detection of miR-338-3p expression in normal breast cells and breast cancer cell lines. Statistical significance indicated as ***P* < 0.01, ****P* < 0.001, *****P* < 0.0001. (B) Analysis from the METABRIC dataset showing the relationship between miR-338-3p expression and prognosis of breast cancer patients. (C) Wound-healing experiments conducted in miR-338-3p mimic-treated breast cancer cell lines MDA-MB-231 and MCF7. Statistical significance indicated as ***P* < 0.01, ****P* < 0.001. The experiment was repeated three times. (D, E) Wound-healing experiments performed on breast cancer cells MDA-MB-231 and MCF7 overexpressing circGSK3β and overexpressing circGSK3β while overexpressing miR-338-3p. Statistical significance indicated as **P* < 0.05, ***P* < 0.01, ****P* < 0.001, *****P* < 0.0001. The experiment was repeated three times.

**Supplementary Figure 5**

**
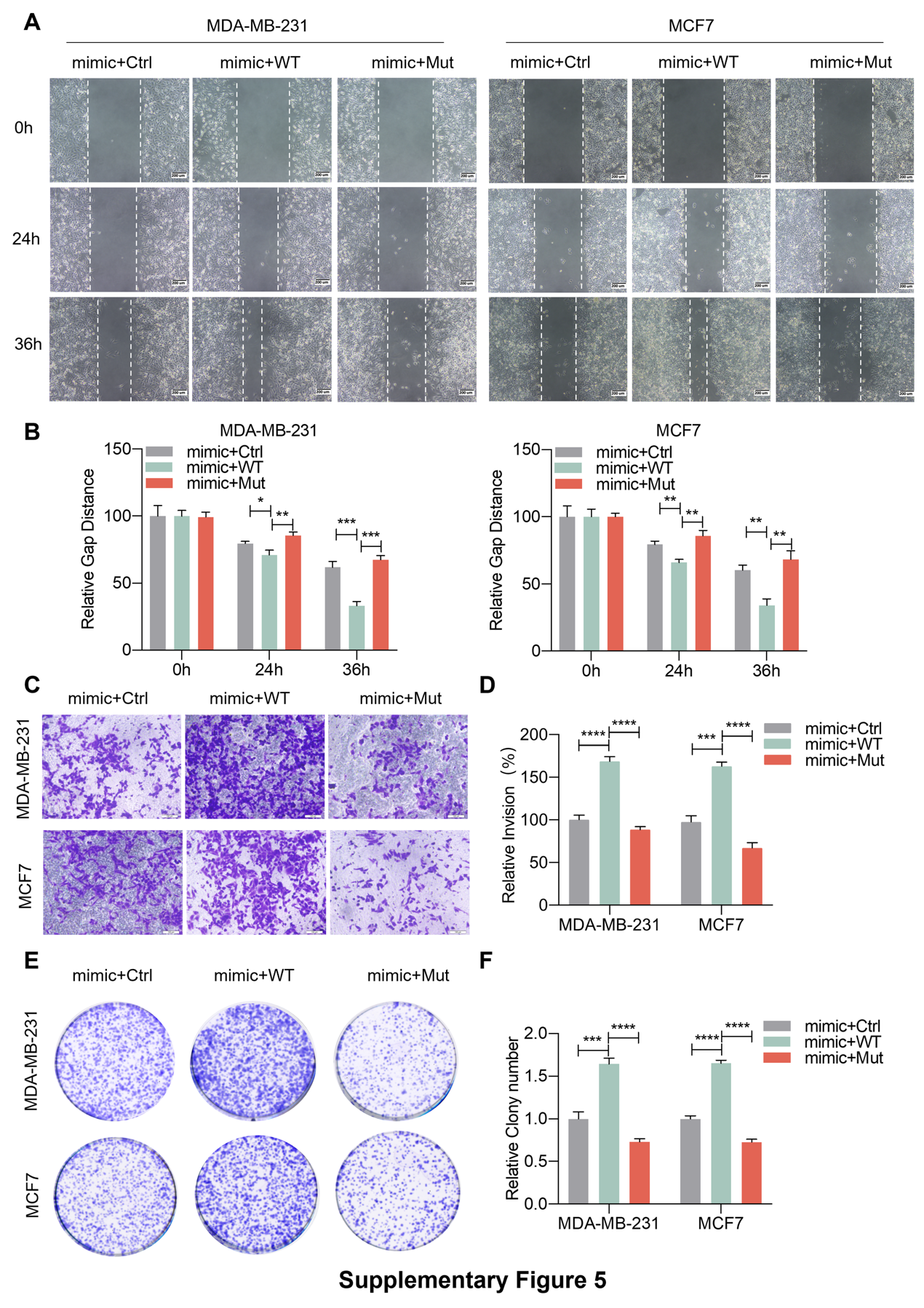
**

**Supplementary Figure 5. The wild-type circGSK3β exerts its function through miR-338-3p.** (A, B) Wound-healing experiments conducted in BC cell lines MDA-MB-231 and MCF7 transfected with empty vector + miR-338-3p mimic (mimic + Ctrl), overexpressing circGSK3β wild-type (mimic + WT), or circGSK3β mutant (mimic + Mut). (A) Representative image of the wound-healing experiment; (B) Statistical analysis of the wound-healing experimen.Statistical significance indicated as **P* < 0.05, ***P* < 0.01, ****P* < 0.001. The experiment was repeated three times. (C, D) Transwell analysis of BC cell lines MDA-MB-231 and MCF7 transfected with empty vector + miR-338-3p mimic (mimic + Ctrl), overexpressing circGSK3β wild-type (mimic + WT), or circGSK3β mutant (mimic + Mut). (C) Representative image of the Transwell experiment; (D) Statistical analysis of the Transwell experiment. Statistical significance indicated as ****P* < 0.001, *****P* < 0.0001. The experiment was repeated three times. (E, F) Colony formation experiments in BC cell lines MDA-MB-231 and MCF7 transfected with empty vector + miR-338-3p mimic (mimic + Ctrl), overexpressing circGSK3β wild-type (mimic + WT), or circGSK3β mutant (mimic + Mut). (E) Representative image of the colony formation experiment; (F) Statistical analysis of the colony formation experiment. Statistical significance indicated as ****P* < 0.001, *****P* < 0.0001. The experiment was repeated three times.

**Supplementary Figure 6**


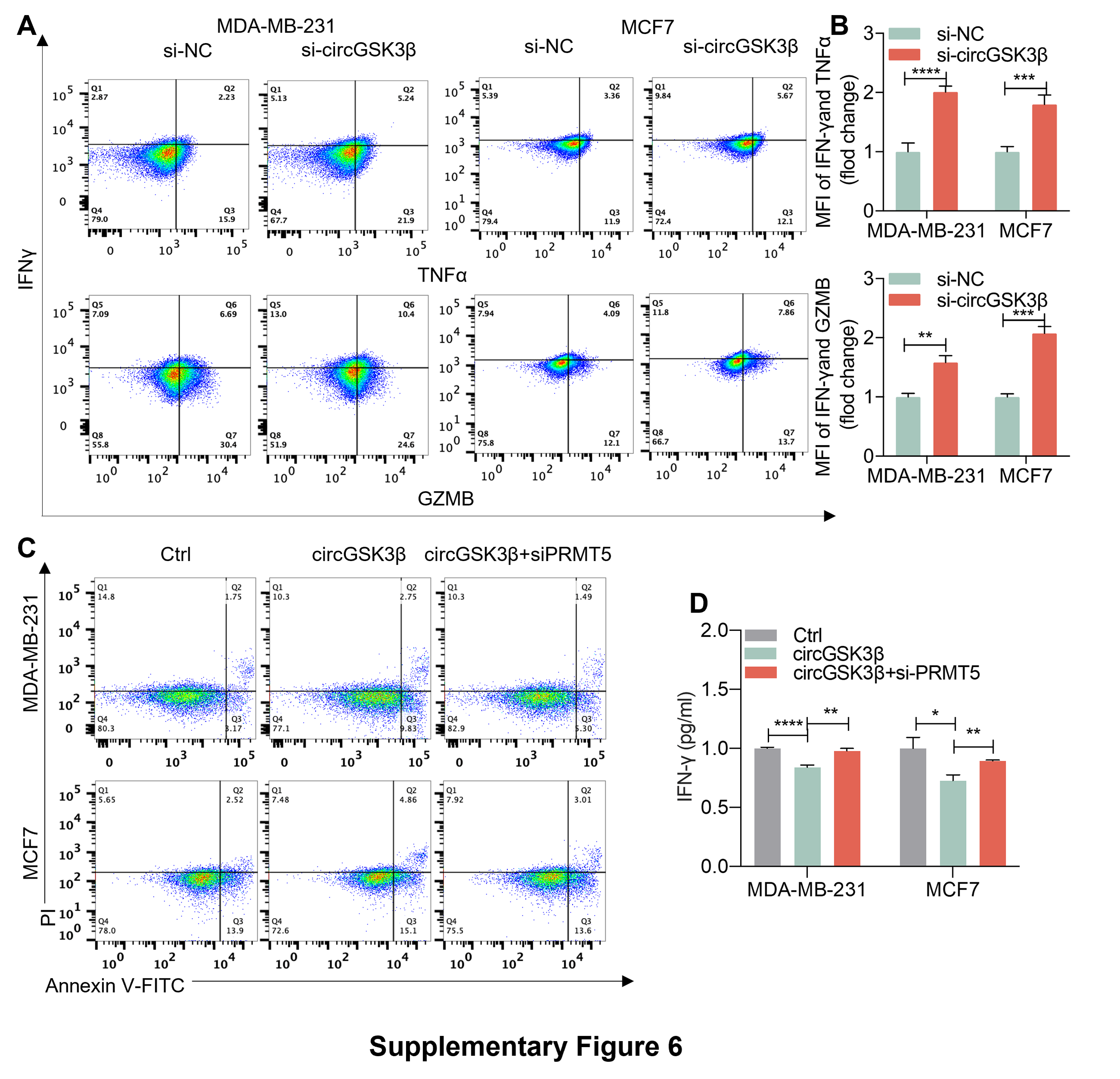


**Supplementary Figure 6. CircGSK3β promotes immune escape and tumor progression through miR-338-3p/PRMT5** .(A, B) Co-culture of breast cancer cells MDA-MB-231 and MCF7 knocking down circGSK3β with T cells, followed by analysis of secretion levels of IFN-γ, TNF-α, and GZMB in primary T cells using flow cytometry. Statistical significance indicated as ***P* < 0.01, *****P* < 0.0001. The experiment was repeated three times. (C, D) Co-culture of breast cancer cells MDA-MB-231 and MCF7 with T cells after overexpressing circGSK3β and overexpressing circGSK3β while knocking down PRMT5, followed by analysis of the apoptosis of primary T cells using flow cytometry.

**Supplementary Table 1. List of siRNAs , miRNA**

| **siRNA** | **Sense（5'-3'）** |
| --- | --- |
| siNC | UUCUUCGAACGUGUCACGUTT |
| si-circGSK3β-001 | TTCACCACTCAAGATGTAT |
| si-circGSK3β-002 | CACTCAAGATGTATGGTCT |
| si-circGSK3β-003 | CAAGATGTATGGTCTGCTG |
| si-PRMT5 | GAGGTGCAGTTCATCATCA |
| miR-338-3p mimic | UCCAGCAUCAGUGAUUUUGUUG |

**Supplementary Table 2. List of qRT-PCR primers**

| **Primers** | **Sense（5'-3'）** |
| --- | --- |
| PD-L1 -Forward | CAATTTGTGCATGGAGAGGAAG |
| PD-L1 -Reverse | GTTGTATGGGGCATTGACTTTC |
| IFN-γ-Forward | TCAGCTCTGCATCGTTTTGG |
| IFN-γ-Reverse | GTTCCATTATCCGCTACATCTGAA |
| IL-2-Forward | AACTCACCAGGATGCTCACATTTA |
| IL-2-Reverse | TCCCTGGGTCTTAAGTGAAAGTTT |
| GZMB-Forward | CTGCTCACTGTTGGGGAA |
| GZMB-Reverse  GSK3β-Forward | TGGGGGATGGGTCTTTTC  ACTAAGGATTCGTCAGGAACAGG |
| GSK3β-Reverse | GTGGCATTTGTGGGGGTTGA- |
| PRMT5-Forward | TGCAGTGGCTCTTGAAATTG |
| PRMT5-Reverse | ATGAGCCTCTGGTGCATCTT |
| miR-338-3p-Forward | TCCAGCATCAGTGATTTTGTTG |
| miR-338-3p-Reverse | Universal Primers |
| 18S-Forward | TCTTAGCTGAGTGTCCCGCG |
| 18S-Reverse | ATCATGGCCTCAGTTCCGAA |
| GAPDH-Forward | CGACCACTTTGTCAAGCTCA |
| GAPDH-Reverse | ACTGAGTGTGGCAGGGACTC |

**Supplementary Table 3. List of primary antibodies for western blotting, immunofluorescence, flow cytometry, immunoprecipitation**

| **Antibody** | **SOURCE** | **IDENTIFIER** | |
| --- | --- | --- | --- |
| PRMT5 (D5P2T) Rabbit mAb | Cell Signaling Technology | | 79998 |
| PD-L1 (E1L3N®) XP® Rabbit mAb | Cell Signaling Technology | | 13684 |
| Tri-Methyl-Histone H3 (Lys4)/H3K4me3 Antibody | Affinity Biosciences | | DF6935 |
| HRP Conjugated Anti-GAPDH Recombinant Rabbit Monoclonal Antibody | HUABIO | | ET1702-66 |
| BV421 Mouse Anti-Human CD3 Monoconal antibody | BD Pharmingen | | SK7 |
| PE-CyTM7 Mouse Anti-Human CD8 Monoconal antibody | BD Pharmingen | | RPA-T8 |
| FITC anti-human/mouse Granzyme B Recombinant | BD Pharmingen | | 372206 |
| PE anti-human tnf | BD Pharmingen | | 554513 |
| PERCP-CY5.5 ANTI-HUMAN IFN-R | BD Pharmingen | | 502526 |

**Supplementary Table 4. List of CHIP qRT-PCR primers**

| **Primers** | **Sense（5'-3'）** |
| --- | --- |
| PD-L1-1-Forward | ACCAATGCAAGGGCTATCTC |
| PD-L1-1-Reverse | CTCAAGTGATCCGCCAAAGT |
| PD-L1-2-Forward | ACTTTGGCGGATCACTTGAG |
| PD-L1-2-Reverse | GTTAGTGAATGGGCCCAAGA |
| PD-L1-3-Forward | TCTTGGGCCCATTCACTAAC |
| PD-L1-3-Reverse | AACTTCCCATCCCGAGCTAC |
| PD-L1-4-Forward | CTCGGGATGGGAAGTTCTTT |
| PD-L1-4-Reverse | GACCCATATGGCTTTGGTTTT |
| PD-L1-5-Forward | AAAGCCATATGGGTCTGCTG |
| PD-L1-5-Reverse | CAACATCTGAACGCACCTTG |
| PD-L1-6-Forward | CAAGGTGCGTTCAGATGTTG |
| PD-L1-6-Reverse | TTTTCACCGGGAAGAGTTTC |
| PD-L1-7-Forward | CTTCCCGGTGAAAATCTCAT |
| PD-L1-7-Reverse | CAAGGCAGCAAATCCAGTTT |
