# Supplementary figures and images for: CircGSK3β mediates PD-L1 transcription through miR-338-3p/PRMT5/H3K4me3 to promote breast cancer cell immune evasion and tumor progression
Source: Cell Death Discov. 2024 Oct 4;10:426. doi: 10.1038/s41420-024-02197-8 (PMC11452702; doi:10.1038/s41420-024-02197-8)

Figure 4C

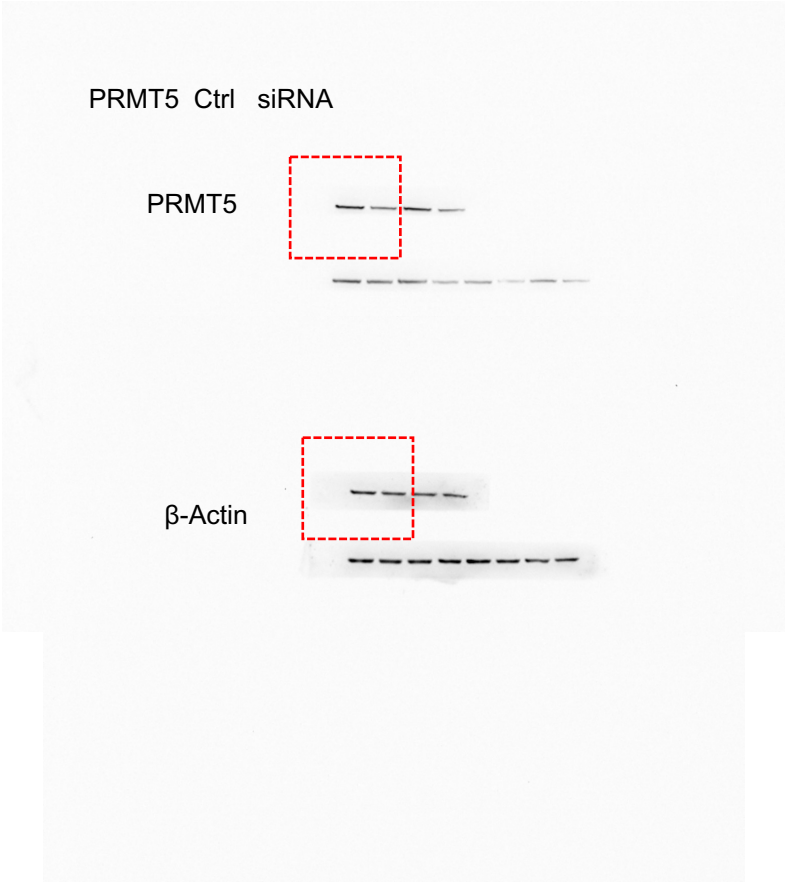

Figure 4F

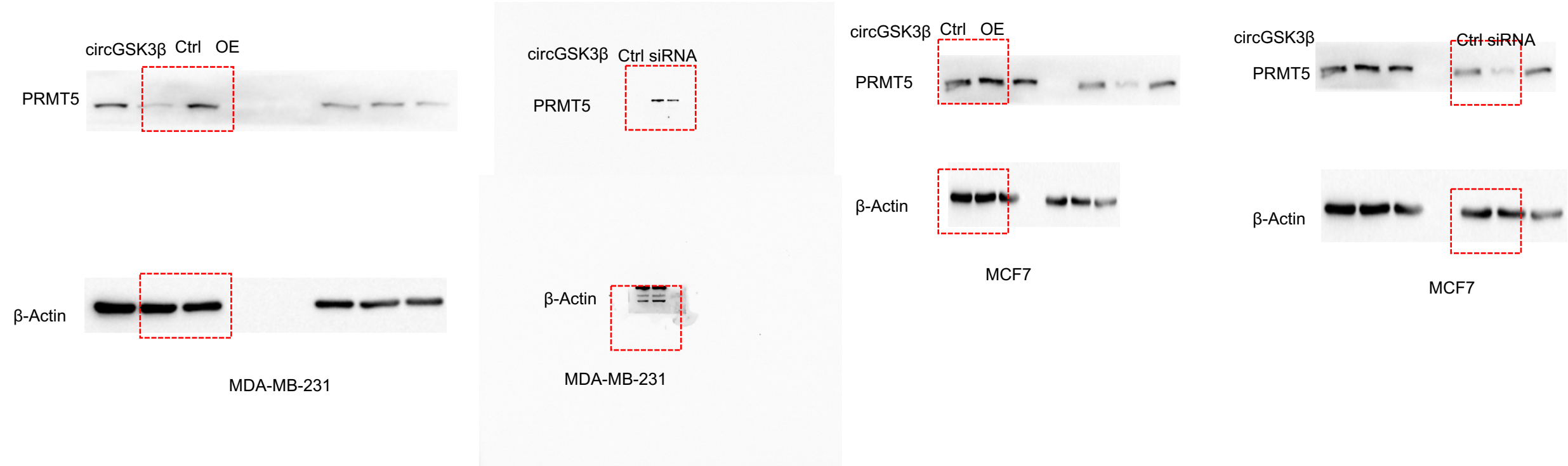

Figure 4G

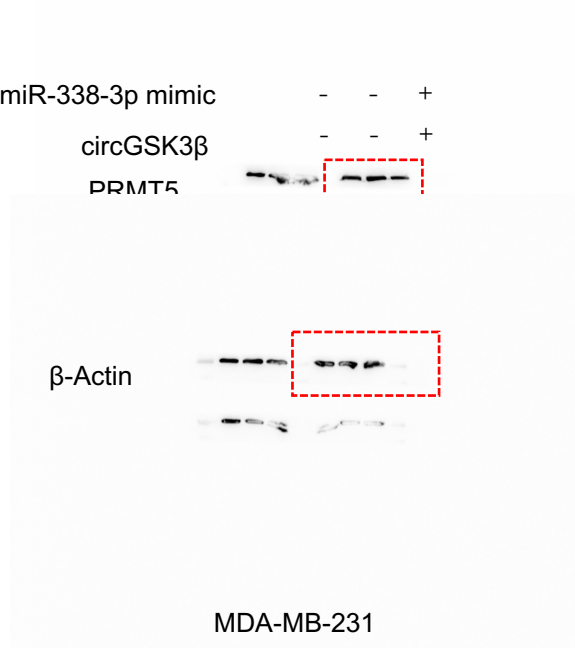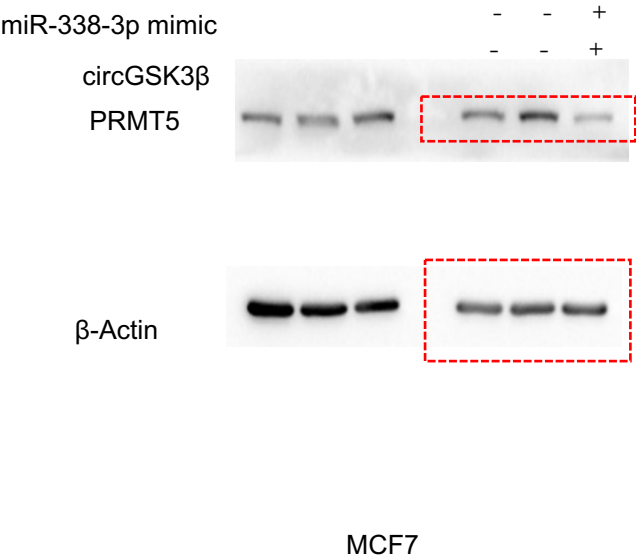

Figure 4I

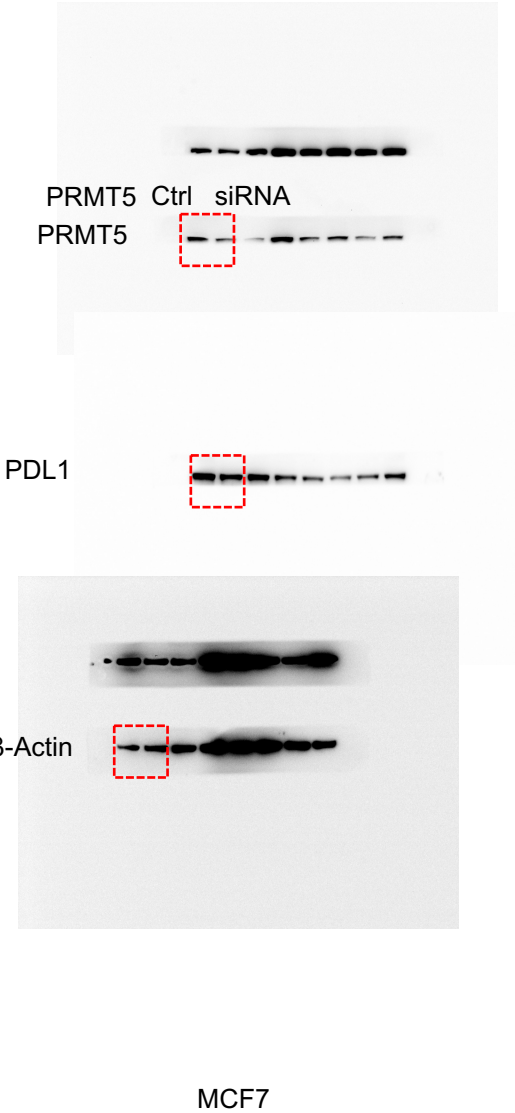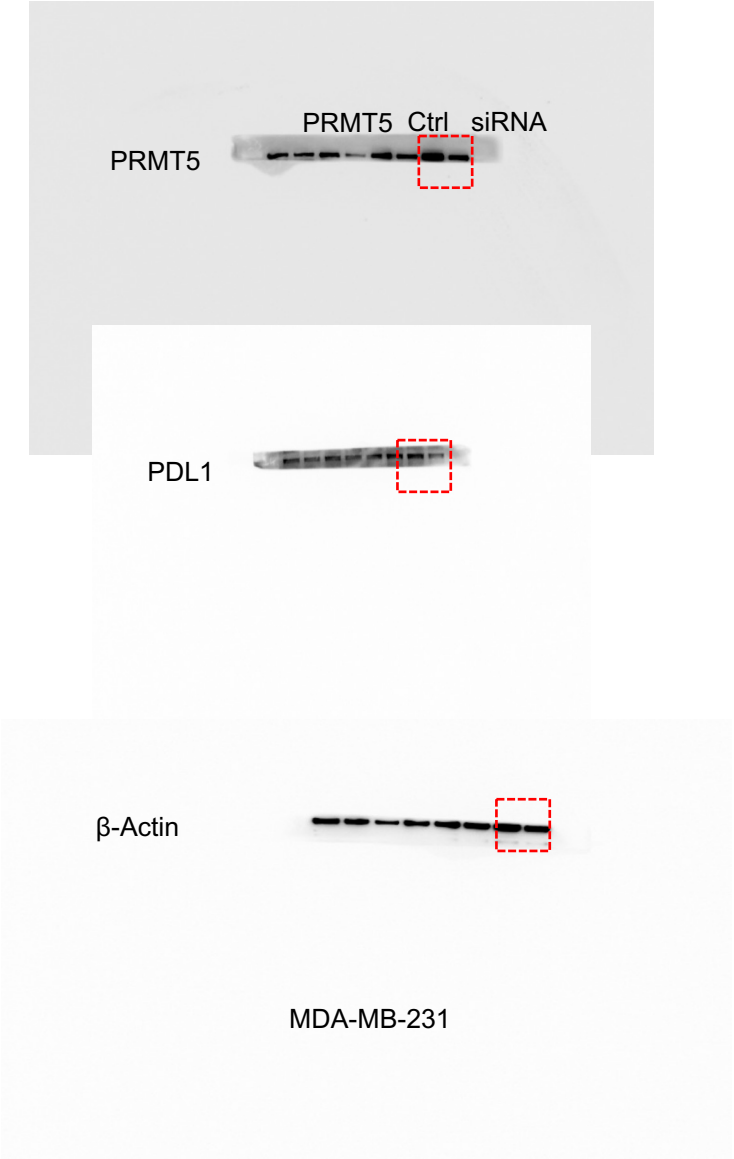

Figure 4J

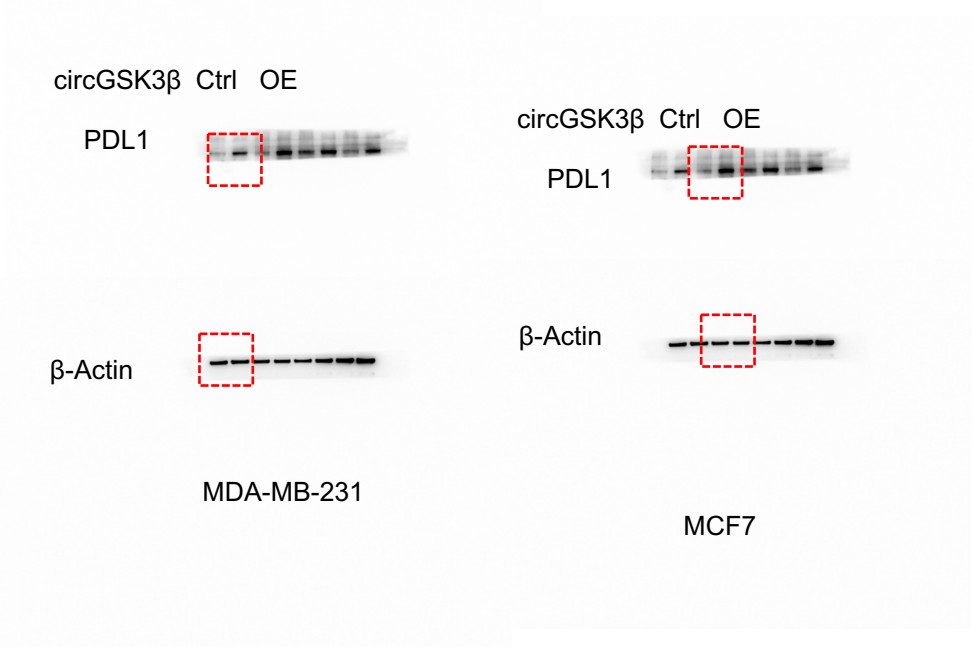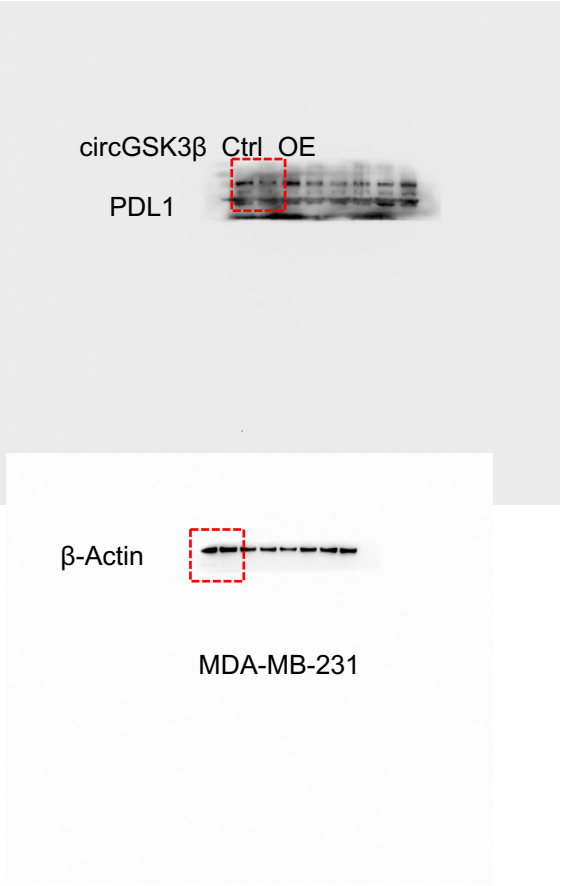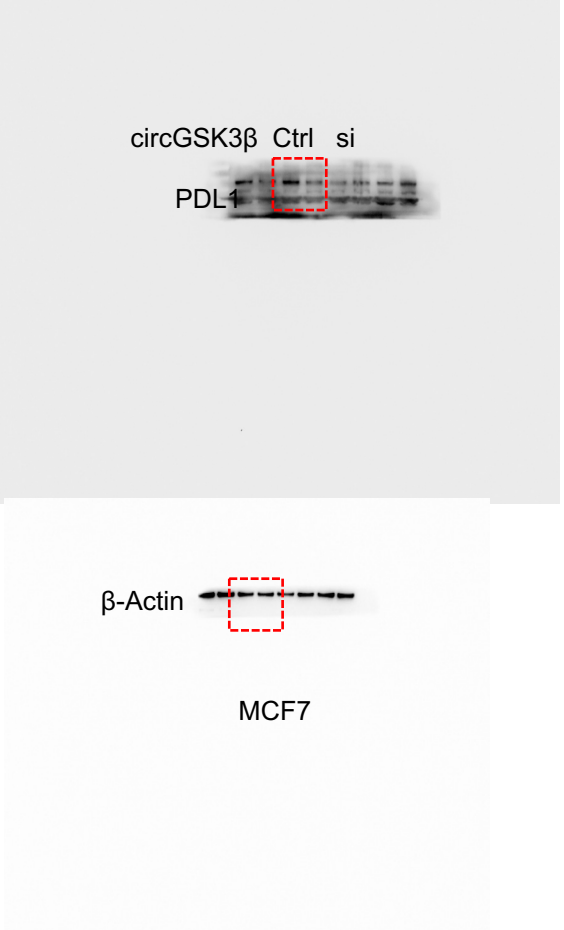

Figure 5F

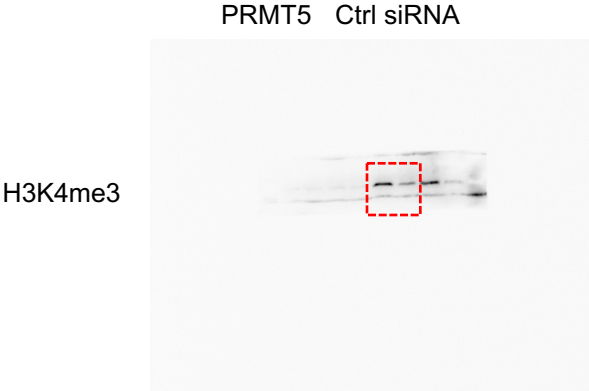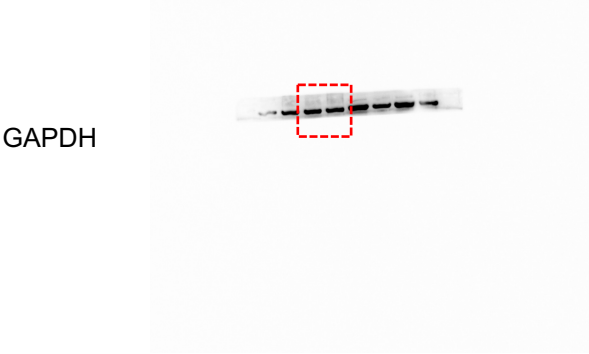

MDA-MB-231

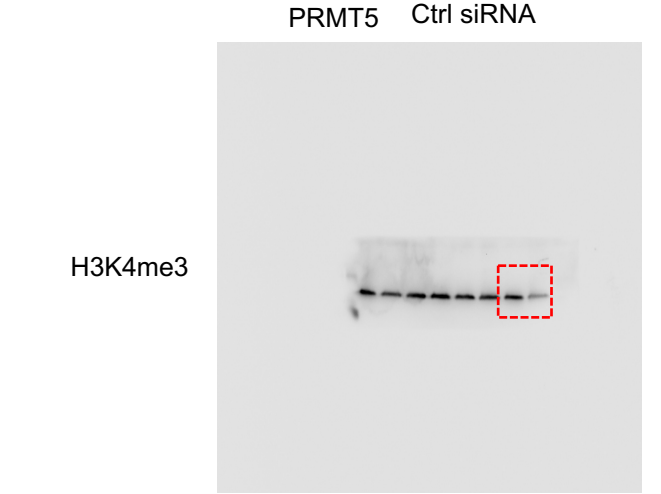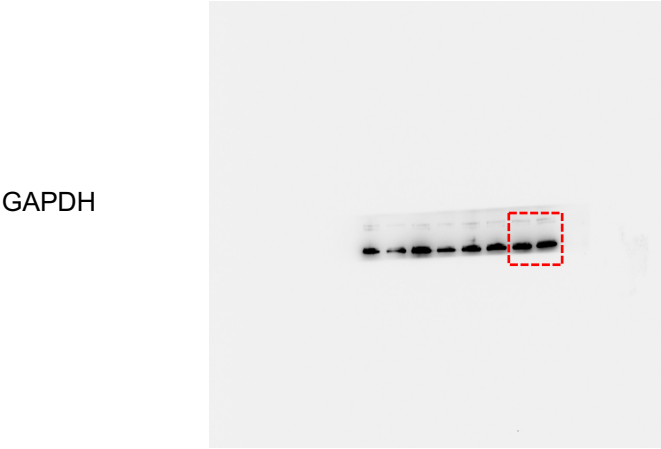

MCF7

Supplement: Supplementary file 2 — wb image [file 41420_2024_2197_MOESM2_ESM.pdf]
